# Solvent-Mediated Phase Propagation Governs Excitonic Heterogeneity in Chiral Fibrils

**DOI:** 10.1021/acscentsci.6c00969

**Published:** 2026-08-28

**Authors:** Philip Daniel Maret, Brijith Thomas, Mahesh Hariharan

**Affiliations:** † School of Chemistry, 193159 Indian Institute of Science Education and Research (IISER) Thiruvananthapuram, Kerala 695551, India; ‡ Science Division, 167632 New York University Abu Dhabi, 129188 Abu Dhabi, United Arab Emirates

## Abstract

Phase propagation and phase separation in biomolecular condensates have emerged as key concepts for understanding the nonequilibrium evolution of functional assemblies, yet achieving analogous processes in soft molecular aggregates remains challenging. Here, we directly visualize phase propagation between coexisting J-type coupled and excimer-emitting domains within individual crystalline fibrils of a chiral perylenediimide dimer (RCy-PDI_2_) using fluorescence lifetime imaging microscopy (FLIM). FLIM analysis revealed heterogeneous distribution of short (τ_*blue*_ ≈ 1.5 ns) and long (τ_*green*_ ≈ 8 ns) fluorescence lifetime components in a single aggregate fibril. Time-dependent FLIM measurements facilitated the understanding of phase propagation and shrinkage-induced fibril fracture in the aggregate fibrils. Steady-state and chiroptical measurements of (RCy-PDI_2_) aggregate fibrils indicated the presence of J-type aggregate formation and excimer emission. Three-dimensional electron diffraction (3D ED) reveals a toluene-intercalated structure enforcing a V-shaped geometry (interplanar angle: 26.3°), enabling strong Coulombic coupling (*J*_c_ = -55 to -73 meV) consistent with J-type interactions. Toluene diffusion and detrapping drive structural relaxation, converting J-type domains into excimer-emitting regions within the same fibril, establishing a direct structural basis for excitonic state selection. This work correlates excitonic heterogeneity with phase propagation, expanding our understanding of the role of solvent diffusion and molecular stacking in the development of functional supramolecular materials.

## Graphical abstract

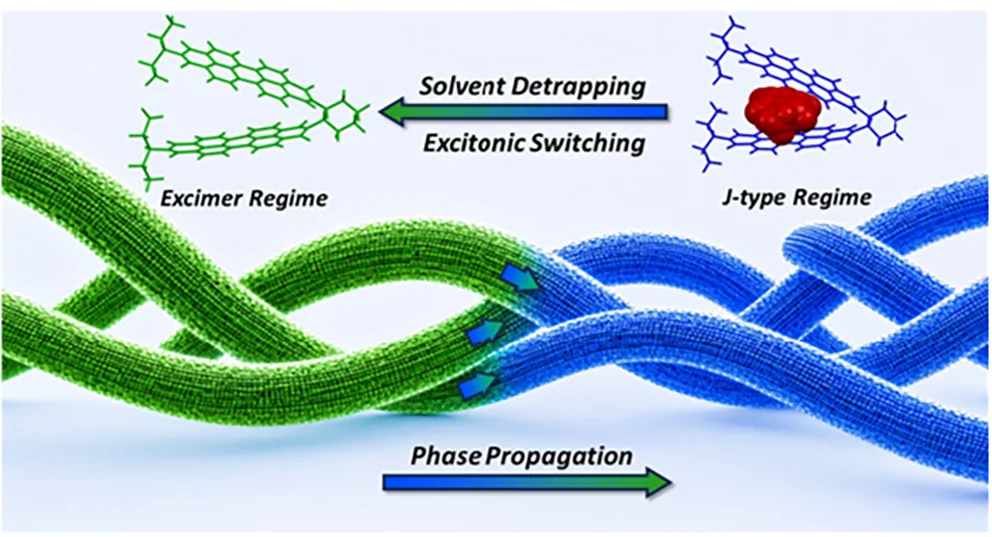

## Introduction

Phase propagation is a fundamental nonequilibrium phenomenon governing structural evolution across a broad range of biological,^1,2^ molecular,^3,4^ and condensed-matter systems.^5^ In biological assemblies, propagative structural transitions drive the maturation of amyloid fibrils,^6^ α-synuclein condensates,^7^ and cytoskeletal architectures through cooperative spatial arrangement.^3^ Similarly, propagating phase boundaries dictate the polymorphic evolution, domain growth, and structural transformations in crystalline solids, ferroic materials,^8^ polymers, and multicomponent alloys.^9^ Unlike homogeneous phase transformations that occur uniformly throughout a material, propagative transformations proceed via the motion of an interface separating structurally distinct phases. Understanding the molecular origin and kinetics of such propagating phase boundaries is therefore essential for controlling material heterogeneity and emergent functionality.

Crystalline assemblies of π-conjugated chromophores are particularly sensitive to local variations in molecular packing, solvent occupancy, and defect density, often producing coexisting excitonic domains with fundamentally distinct optical properties.^10,11^ Small structural perturbations within these aggregates, substantially alter intermolecular electronic coupling and excited-state dynamics, resulting in heterogeneous photophysical behavior.^12^ Such heterogeneity frequently originates from solid-state phase transformations triggered by external stimuli including solvent environment, temperature, pH, and/or mechanical perturbation.^13^ Phase transformations in supramolecular systems were widely investigated through phenomena such as single-crystal-to-single-crystal transitions,^14,15^ supramolecular polymorphism,^16^ and solvent-mediated structural reorganization.^17^ However, the mechanistic origin and observation of the spatial propagation of phase boundaries within molecular aggregates remain elusive.

Herein, we structurally, spatiotemporally, and spectroscopically map the phase propagation dynamics in fibrillar aggregates of a chiral perylenediimide dimer, RCy-PDI_2_. The RCy-PDI_2_ aggregates formed in toluene exhibit periodic striped domains comprising coexisting J-type aggregate and excimer-emissive states, which were visualized using fluorescence lifetime imaging microscopy (FLIM).^18,19^ Time-dependent FLIM measurements reveal a propagating phase front within individual fibrils, in which the excimer domain progressively expands and consumes the J-aggregate domain via a moving phase boundary. Different aggregate fibrils exhibited distinct phase-front propagation velocities and birefringence, suggesting anisotropic diffusion and optical anisotropy, respectively. To uncover the structural origin of the phase transformation, we employed three-dimensional electron diffraction (3D ED), which provides molecular crystal structures of submicron crystallites that are inaccessible to conventional crystallographic methods.^20,21^ The diffuse distribution of toluene observed in the 3D ED measurements suggests anisotropic solvent diffusion and detrapping as the origin of the propagative phase transformation within the fibrils. The 3D ED analysis further reveals that a solvent-stabilized geometry promotes strong long-range Coulombic coupling favoring J-type excitonic interactions consistent with the steady-state spectroscopic measurements. The excited-state characteristics were further validated using time-dependent density functional theory (TDDFT) calculations and excitation energy transfer (EET) coupling analysis, yielding intermolecular Coulombic coupling strengths (J_c_) ranging from -55 to -73 meV, consistent with J-type aggregation. Noncovalent interaction analysis further reveals enhanced intermolecular interactions upon solvent evaporation, driving intermolecular excimer formation. To our knowledge, this represents the first direct visualization of a propagating excitonic phase boundary within a molecular aggregate (Table S1).

## Results and Discussion

(*1R,2R*) Cyclohexadiamine-based perylenediimide dimer (RCy-PDI_2_) was synthesized using the previously reported procedure (Scheme S1).^22^ At higher concentrations of RCy-PDI_2_ in toluene (TOL) (above 100 μM), RCy-PDI_2_ molecules tend to assemble into a fibril structure in the mother liquor (Figure 1a). Interestingly, the aggregate fibrils of RCy-PDI_2_ showed a heterogeneous band-like pattern under an optical microscope (60× magnification). Fluorescence lifetime imaging was performed for the aggregate fibrils using a 512 nm excitation laser with a repetition rate of 20 MHz (Figure 1b). Excitation power for sample excitation and imaging was in a few nanowatts range to avoid signal saturation at the single-photon avalanche diode (SPAD) detector. FLIM images of the RCy-PDI_2_ aggregates drop-cast on the coverslip revealed fibers of diverse thicknesses (on the submicron scale) and lengths (from a few micrometres to the millimeter regime). FLIM measurements of the freshly drop-cast RCy-PDI_2_ aggregate fibrils depicted a band-like pattern with distinct phase separation (Figure 1). The fluorescence lifetimes of the RCy-PDI_2_ aggregate fibrils in the FLIM portrayed a short lifetime species (∼1.5 ns, blue phase) and a long fluorescence lifetime species (∼8 ns, green phase). To map the evolution of the green and blue phases in the RCy-PDI_2_ aggregate fibrils, time-dependent FLIM measurements were acquired for a total duration of 270 min with a 30 min interval (Figure 1 and Figure S1). The fluorescence lifetime distribution profile was plotted from 0 to 270 min for the selected FLIM image of the RCy-PDI_2_ aggregate fibrils. A considerable shift in the fluorescence lifetime distribution was observed with the contribution of long lifetime species dominating with time (Figure 1c). Interestingly, when the aggregate fibrils were observed carefully, they showed a phase propagation behavior with the green phase propagating and consuming the blue phase. Few selected RCy-PDI_2_ aggregate fibrils (A, B and C) were monitored in the FLIM images, and phase propagation was visible when transitioning from 0 to 270 min (Figure 1d and 1e). Phase front velocities for the selected RCy-PDI_2_ aggregate fibrils were tabulated as a function of phase migration distance, analyzed using ImageJ software over 270 min (Figure 1f). The phase front velocities differed among the A, B and C fibrils in the FLIM image, suggesting the contribution of anisotropic behavior in the RCy-PDI_2_ aggregate fibril.

**Figure 1:**
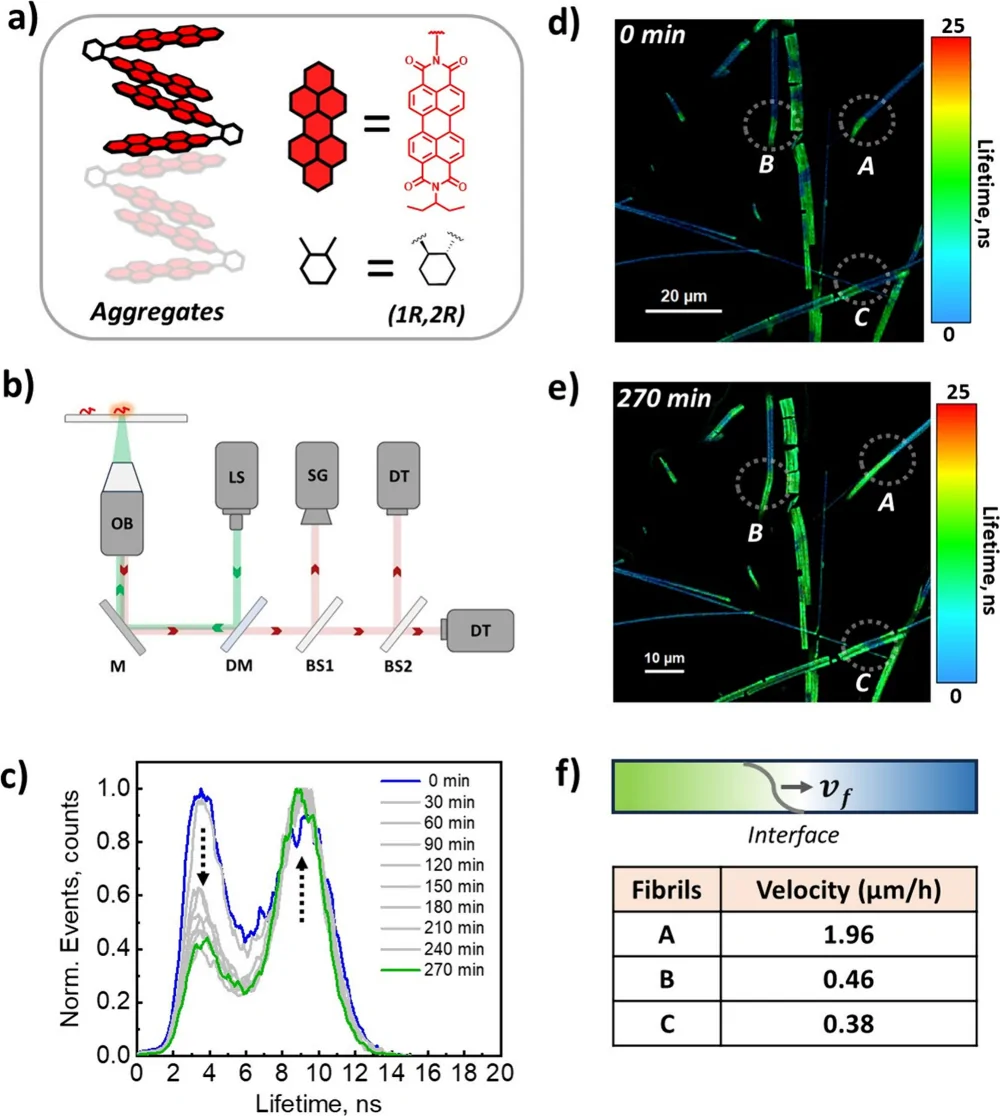
(a) Schematic representation of the aggregation of RCy-PDI_2_ in toluene. (b) Schematic representation of the single-molecule fluorescence microscope used for the FLIM and fluorescence emission measurements. OB: objective, LS: excitation laser, DT: detector, SG: spectrograph, BS1:50:50 beam splitter, BS2:50:50 beam splitter, DM: dichroic mirror and M: mirror. The excitation laser is depicted in green, and the emission light is shown in red. The arrowheads indicate the laser’s path. (c) The switching of the fluorescence lifetime distribution with time is measured from zero minutes to 270 min with a 30 min interval. FLIM images of the aggregate fibrils at (d) 0 min and (e) 270 min with the fibrils showing phase propagation highlighted as A, B and C. (f) Phase front velocity of the aggregate fibrils A, B, and C are highlighted.

To decipher the nature of the excitonic states within the aggregate fibrils, FLIM combined with the spectrograph was used to characterize the observed phases in the RCy-PDI_2_ aggregates on a neat film (Figure 2a). Scanning electron microscopy (SEM) images of the RCy-PDI_2_ aggregates revealed a fibrillar structure exhibiting negligible heterogeneity (Figure 2b). To obtain high-quality FLIM images, the dwell time was set to a range of 0.4 - 0.6 ms to increase fluorescence counts. Pseudolifetime color coding in the FLIM images indicated the existence of two diverse species in the RCy-PDI_2_ aggregates (Figure 2a and Figure S2). Fluorescence lifetime measurements for the RCy-PDI_2_ aggregates from the overall FLIM image showed a biexponential decay profile, fitted with fluorescence lifetimes of τ_*green*_ ≈ 8 ns and τ_*blue*_ ≈ 1.5 ns, respectively. Upon selective photoexcitation of the blue and green phases observed in the FLIM image (Figure 2a), a nearly monoexponential decay profile was obtained from the selected aggregate fibril domains (Figure 2c). The short fluorescence lifetime component (τ_*blue*_ ≈ 1.5 ns) of RCy-PDI_2_ aggregates relative to the monomer fluorescence lifetime (τ_*m*_ ≈ 2.8 ns) of RCy-PDI_2_, is consistent with emission from J-type coupled domains formed by RCy-PDI_2_ molecules within the aggregate fibril.^10,23^ On the other hand, a long fluorescence lifetime component (τ_*green*_ ≈ 8 ns) relative to the monomer in the aggregate fibril possibly arises from excimer formation in the RCy-PDI_2_ aggregate fibers.^24^

**Figure 2:**
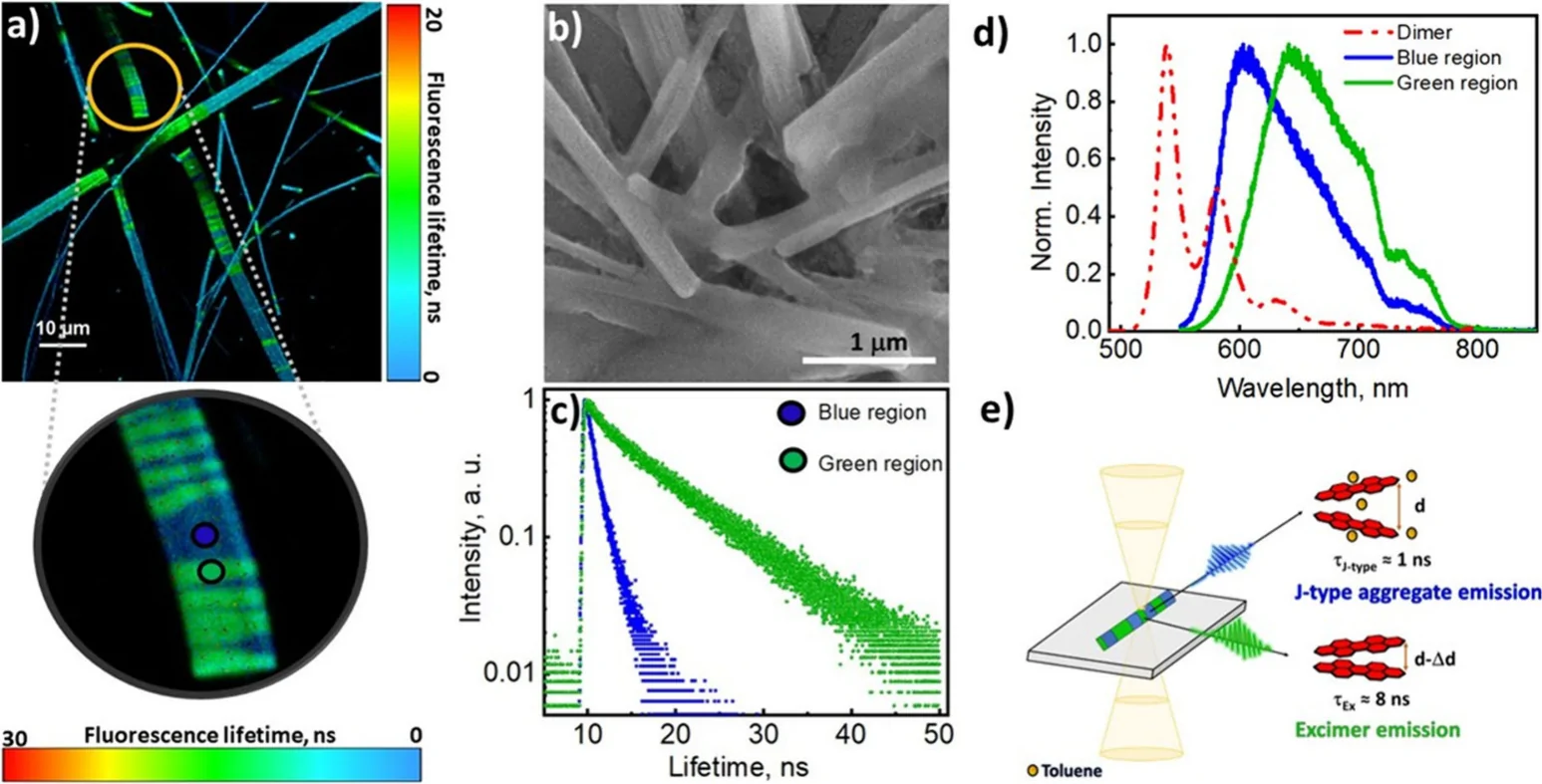
(a) Fluorescence lifetime image of the RCy-PDI_2_ aggregate fibers on a coverslip with a zoomed-in version of one of the striped aggregates. The blue and green spots are markers of the region from which the fluorescence lifetime decay was obtained. (b) SEM image of the aggregate fibers (in toluene) obtained by drop-casting a 500 μM RCy-PDI_2_ solution. (c) TCSPC decay profiles of the point traces obtained for the blue and the green region of the fiber. (d) The fluorescence emission spectra acquired from the depicted blue and green regions using a spectrograph. The dimer emission corresponds to the solution state emission measurement of RCy-PDI_2_. (e) The schematic representation of the corresponding excited state responsible for the emission and the structural alteration triggering the heterogeneity is also proposed.

To deconvolute the emissive states of the RCy-PDI_2_ aggregates, fluorescence emission spectra were collected with a spectrograph from the blue (short fluorescence lifetime) and green (long fluorescence lifetime) regions of the aggregate fibers (Figure 2d and Figure S3). The fluorescence emission spectrum of the nonaggregate solution of RCy-PDI_2_ in toluene spans from 500 to 700 nm. The fluorescence emission spectrum from the blue region (based on FLIM) of the RCy-PDI_2_ aggregate fibril exhibits a bathochromic shift relative to the RCy-PDI_2_ monomer (Δυ _monomer‑blue_ ≈ 1942 cm^–1^). Whereas the emission spectrum from the green region is further bathochromically shifted (Δυ_monomer‑green_ ≈ 2952 cm^–1^) from the RCy-PDI_2_ monomer (Table S2). Fluorescence emission spectrum from the green region of the RCy-PDI_2_ aggregate fiber depicted a broad spectral profile with a peak maximum around 640 nm. The substantial spectral line width indicates that J-type excitonic coupling coexists with energetic disorder and limited exciton delocalization, placing the system in a nonideal excitonic regime distinct from canonical J-aggregates.^25^ The distinct emission profiles for the blue and green regions of the RCy-PDI_2_ aggregate strand corroborate the presence of a J-type aggregate and excimer emission, respectively (Figure 2d). To further spectroscopically characterize the RCy-PDI_2_ aggregate, concentration-dependent steady-state absorption (Figure S4a) and emission (Figure S4b) measurements were performed in TOL under ambient conditions. Upon increasing the concentration of RCy-PDI_2_ from 100 μM to 700 μM in TOL, the absorption spectra of RCy-PDI_2_ showed a broad spectral feature developing with a peak maximum at ∼560 nm, which was absent in the RCy-PDI_2_ absorption spectra reported for monomer concentrations in TOL solution (Figure S4a). Since the diverse aggregate bands are observed in the solid RCy-PDI_2_ aggregate fibrils, the Kubelka–Munk transformed diffuse reflectance spectrum was measured for a neat film sample cast on a glass coverslip (Figure 3a). Solid-state electronic absorption spectrum for RCy-PDI_2_ in neat film depicted a peak at ∼562 nm and a shoulder band at ∼505 nm. The bathochromic shift in the solid-state absorption spectrum of RCy-PDI_2_ relative to monomer solution-state measurements of RCy-PDI_2_ in TOL corroborates J-type excitonic coupling in RCy-PDI_2_ aggregates.^25–27^ Solution state fluorescence emission spectra of RCy-PDI_2_ were recorded at concentrations ranging from 100 μM to 600 μM. The decrease in fluorescence intensity observed for the first vibronic band in the emission spectrum of RCy-PDI_2_ aggregates in TOL possibly stem from the inner-filter effect,^28^ which is prominent at optical densities above 0.05. To access the intrinsic emissive states of the aggregate free from inner-filter contributions, solid-state fluorescence measurements were performed on a dry, neat RCy-PDI_2_ aggregate film (Figure 3b). Compared with the solution-state fluorescence measurements for RCy-PDI_2_ in TOL, a broad and red-shifted emission was observed, possibly indicating multiple emissive states (such as excimer and aggregate emission).^24^ Since the RCy-PDI_2_ aggregate was formed from a chiral PDI dimer, understanding the aggregate chirality is crucial for comprehending how RCy-PDI_2_ assemblies may function in chiroptical applications.^29^

**Figure 3:**
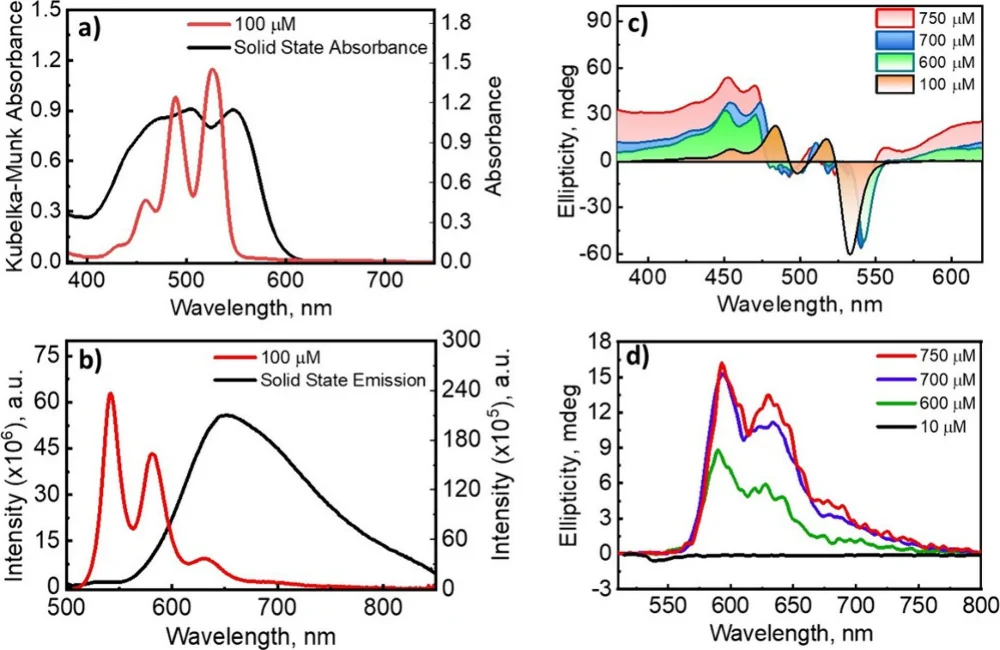
(a) The steady-state absorption measurements of RCy-PDI_2_ in solution (toluene) and solid state (neat film). The aggregate formed was drop-cast on the coverslip and used for the solid-state measurement. The band around 562 nm is characteristic of the aggregation. (b) The solution-state fluorescence spectrum at 100 μM concentration along with the solid-state fluorescence spectrum of the aggregate. (c) The concentration-dependent CD spectra of RCy-PDI_2_ aggregate in toluene. (d) Concentration-dependent circularly polarized luminescence measurements of RCy-PDI_2_ in toluene.

To probe the evolution of chirality in RCy-PDI_2_ aggregate fibrils formed in TOL, concentration-dependent circular dichroism (CD) measurements were carried out for RCy-PDI_2_ in TOL (Figure 3c). The intense peak at 543 nm from the CD spectra for RCy-PDI_2_ above 100 μM, showed a red-shift (Δλ_monomer–aggregate_ ≈ 9 nm) and a narrow full-width half-maximum (fwhm: 10 nm) compared to the CD spectrum of RCy-PDI_2_ solution at 100 μM (fwhm: 15 nm). The CD vibronic peak for RCy-PDI_2_ at 485 nm in 100 μM is blue-shifted to 471 nm at higher concentrations. The red-shift and narrow fwhm at 543 nm in the RCy-PDI_2_ CD spectra above 100 μM are also consistent with J-type excitonic interactions, although the substantial line width indicates a nonideal, disordered excitonic regime.^30^ The blue-shift in the CD peak at 485 nm possibly arises from the enhanced structural rigidity due to aggregate formation resulting in higher energy vibronic transitions. Chiral supramolecular aggregates of perylenediimides usually exhibit enhanced chiral emission, which depends on the excitonic interactions in the aggregate.^31^ Circularly polarized luminescence (CPL) measurements were carried out for RCy-PDI_2_ in TOL at concentrations of 10 μM, 600 μM, 700 μM, and 750 μM (Figure 3d). The RCy-PDI_2_ sample was excited at 480 nm to obtain CPL spectra spanning from 550 to 800 nm. Interestingly, the aggregated solutions of RCy-PDI_2_ exhibited enhanced emission and a reversal in the sign of the CPL signal compared to the dimer emission at lower concentrations. The g_lum_ value for the aggregated solution of RCy-PDI_2_ was evaluated to be ∼2.02 × 10^–2^ at 593 nm, which is nearly 7-fold higher in magnitude than the reported value for the dimer concentration (reported for dimer in TOL, g_lum_ = -3 × 10^–3^).^22^ Enhancement in g_lum_ value with fiber-like morphology was also reported for a biphenyl-bridged perylenediimide dimer aggregate, with a g_lum_ value twice that of the spherical aggregate analogue and 10 times more enhanced than the monomer, which is in accordance with the present work.^32^ Observed sign inversion in the CPL emission for RCy-PDI_2_ in TOL is probably due to differences in the emission anisotropy contributions from the aggregate compared to the dimer.^33^ A precise understanding of the morphology of the RCy-PDI_2_ aggregates was obtained using SEM and transmission electron microscopy (TEM), performed on aggregate fibrils drop-cast onto a silicon wafer and carbon tape, respectively. Fiber-like structures of RCy-PDI_2_ molecules with lateral dimensions ranging from a few nanometers to micrometers were observed from the SEM images of the drop-cast sample (Figure S5). Time-dependent SEM measurements were conducted on the RCy-PDI_2_ aggregates to monitor the morphological dynamics during aggregate formation (Figure S6). Aliquots acquired at different time intervals from the RCy-PDI_2_ aggregate solution were drop-cast onto a silicon wafer, dried under vacuum, and stored in a desiccator. The aliquots taken from the RCy-PDI_2_ aggregate solution after 2 h show small fibrils with the lateral dimensions in the nanometer regime. Notably, the RCy-PDI_2_ aggregate sample collected after 12 h depicts higher superstructures with lateral dimensions in the micrometer regime (Figure S6). Additionally, TEM measurements of the drop-cast RCy-PDI_2_ aggregates further confirm that small fibrillar aggregates are initially formed, which bundle up with time to form higher superstructures (Figure S7 and Figure S8). Optical images of the RCy-PDI_2_ aggregate fibrils at higher magnification (100×) depicted different brightness when examined through the bright field mode, which possibly suggests the distinct refractive indices in different segments of the RCy-PDI_2_ aggregate fibrils (Figure S9). The polarization optical microscopy images also depicted birefringence, indicating optical anisotropy in the RCy-PDI_2_ aggregate fibrils (Figure S10).

To investigate whether toluene plays a crucial role in aggregate formation, the FLIM images of the RCy-PDI_2_ aggregate fibrils were recorded before and after hot air drying (Figure 4a,c). The overall fluorescence lifetimes from the acquired FLIM images were deconvoluted to the respective components using the FLIM fit feature of the PicoQuant SymPhoTime software. The FLIM images recorded for the fresh RCy-PDI_2_ aggregate before drying show a significant contribution from the J-type aggregate (blue) and a few stripes of the excimer region in the observed aggregate fibril. The slow cooling experiments for aggregate formation confirmed that rapid cooling of the hot supersaturated solution can result in a kinetically trapped random distribution of blue and green phases (Figure S2). Whereas, the slow cooling of the aggregate fibrils gives superstructures with nearly complete blue phase formation (Figure S11). Deconvolved fluorescence lifetime distribution in the RCy-PDI_2_ aggregate also shows that the J-type aggregate contribution is dominant in the FLIM image of freshly drop-cast samples (Figure 4b). Upon hot-air drying, most of the J-type aggregate region has transformed into an excimer-emitting region (Figure 4c), as evidenced by the fluorescence lifetime distribution profile (Figure 4d). The RCy-PDI_2_ aggregate fibrils exhibited cracks after the phase transformation to the green phase in most FLIM images. The observation was further validated by calculating the reduced volume after reduction of the toluene molecules in the unit cell (*vide infra*). The phase transformation with toluene detrapping builds stress in the aggregate fibril, resulting in cracks across the aggregate fibrils (Figure 4e). Overall, the experiments point to the crucial role of toluene detrapping for excitonic heterogeneity observed in the FLIM measurements (Figure 4f).

**Figure 4:**
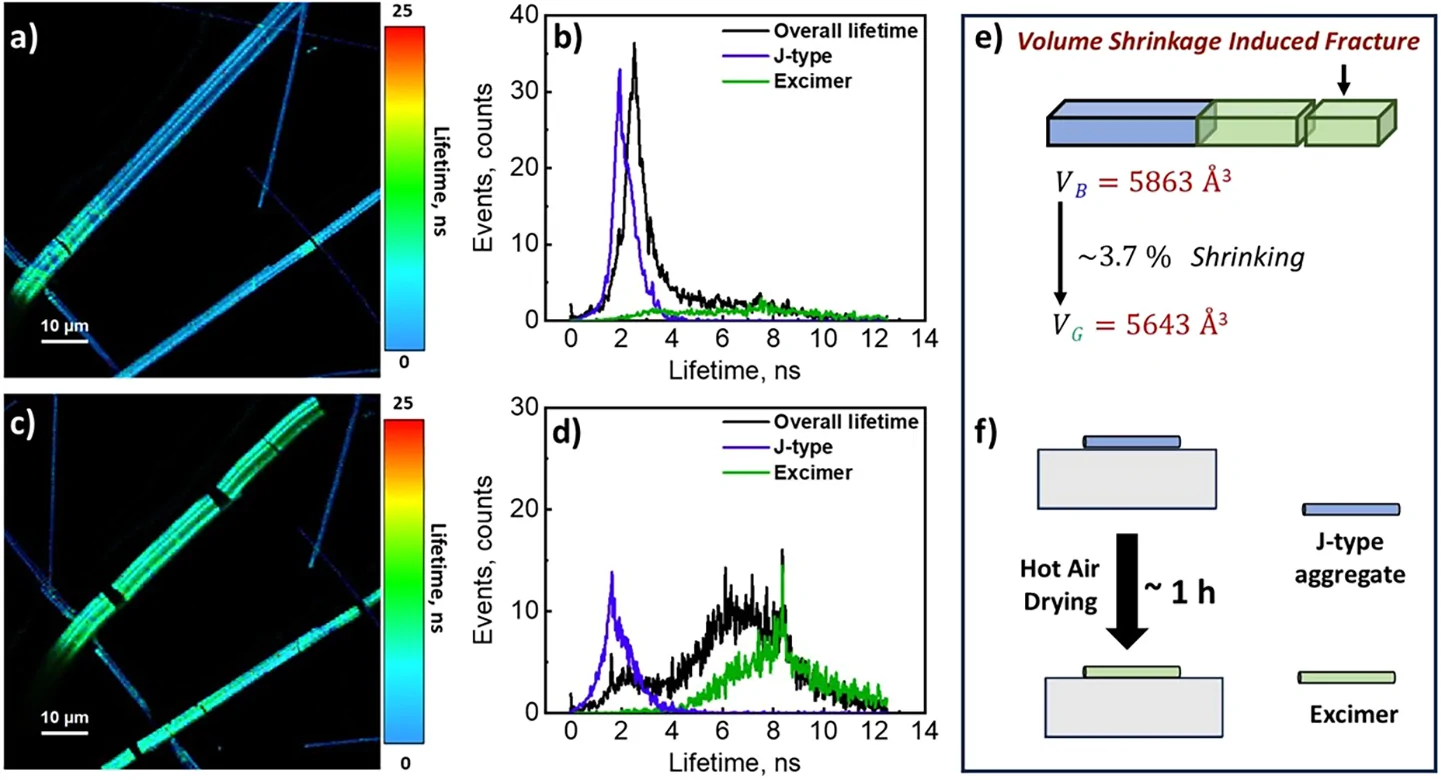
(a) The aggregate fibrils freshly dropcast and imaged using FLIM. (b) The deconvoluted fluorescence distribution profile of the freshly dropcasted aggregate fibril depicts major contribution from the J-type aggregate. (c) The same aggregates upon drying in hot air for 5 min show a significant contribution from the excimer in the FLIM image. (d) The fluorescence lifetime distribution profile for the air-dried sample shows a dominant contribution from the excimer with a fluorescence lifetime of ∼8 ns. (e) The drying results in shrinking and hence crack formation in the aggregate fibril, which was verified from the crystal volume analysis. (f) Schematic procedure of hot air drying experiment of the RCy-PDI_2_ aggregate fibers. After 1 h of drying, the entire J-type aggregate fibril is mostly converted into an excimer-based aggregate.

To elucidate the structure of the RCy-PDI_2_ aggregate and the role of toluene in phase propagation, 3D electron diffraction measurements were performed on submicron fragments of the RCy-PDI_2_ chiral fibrils (Figure 5a and Tables S3–S5). Notably, the resolved structure reveals that a toluene molecule is intramolecularly trapped between the two PDI units (Figure S12). The aggregate structure showed two RCy-PDI_2_ molecules in the asymmetric unit, with one of the dimers containing trapped toluene. The microcrystalline aggregate sample has the *P*2_1_ space group with unit cell parameters of 15.34 Å (a), 18.29 Å (b), 21.29 Å (c), and 90.00° (α), 96.34° (β), and 90.00° (γ). When viewed along the *c*-axis in the aggregate crystal structure, RCy-PDI_2_ molecules are stacked on top of one another with alternating orientations (Figure S12). Necessary constraints on the geometric parameters were applied to determine the RCy-PDI_2_ aggregate structure obtained from 3D ED measurements.

**Figure 5:**
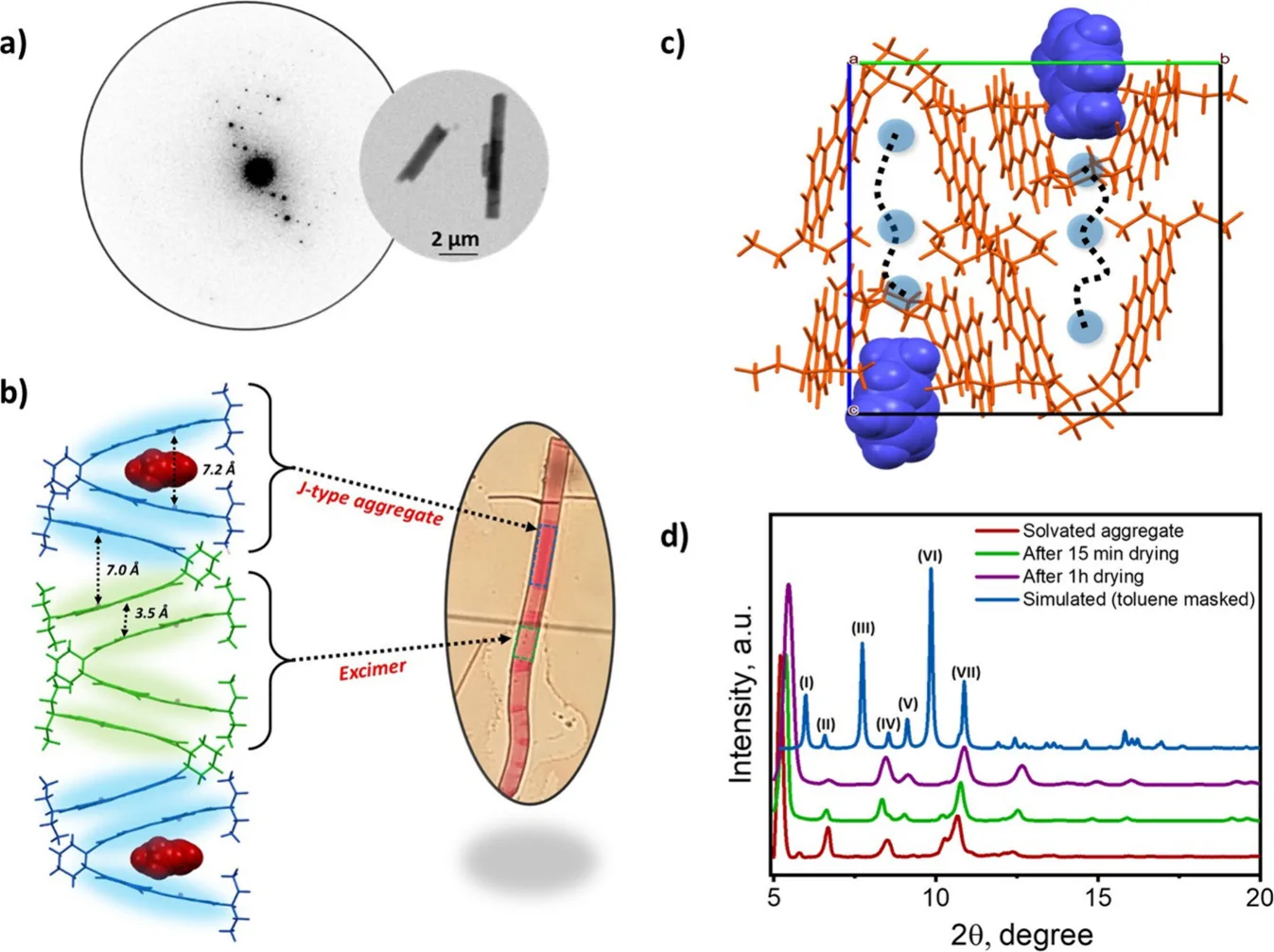
(a) 3D ED measurements for the RCy-PDI_2_ aggregates for a representative aggregate at room temperature. (b) 3D ED crystal structure of the crystalline aggregated fiber of RCy-PDI_2_ with toluene trapped intramolecularly. The blue-highlighted region indicates the PDI units contributing to J-type aggregate formation, and the green-highlighted segment corresponds to the structure dictating the excimer formation. In the optical microscopy image of the aggregate, we can assign the regions corresponding to the toluene-trapped region, responsible for J-type aggregate formation, and the toluene detrapped regions, which contribute to the excimer. The visible refractive index difference in the optical image helps assign the toluene-trapped and detrapped regions. The maroon dummy atoms in the PDI core represent the centroids of the individual PDI fragments. (c) The possible toluene diffusion anisotropy indicated from the 3D ED measurements and the path of the toluene is highlighted in blue dots. (d) PXRD measurements for the aggregates were carried out at various stages of heating and vacuum drying. The simulated PXRD pattern was generated for the toluene-masked crystal structure. (I) (100), (II) (011), (III) (101), (IV) (002), (V) (111), (VI) (020) and (VII) (111).

The RCy-PDI_2_ aggregate structure resolved from the 3D ED measurements exhibits a V-shaped geometry with an interplanar angle of 26.3° (Figure S13), and the intermolecular distance between the neighboring RCy-PDI_2_ molecules in the stack is around ∼3.5 Å. Noncovalent interaction analysis allowed us to map the subtle π–π stacking, side-chain interactions, and dispersive forces that drive the hierarchical organization of aggregates, also revealed a prominent intermolecular π-π interaction along the RCy-PDI_2_ molecular stack (Figure S14).^34^ Furthermore, RCy-PDI_2_ aggregate structure depicts alternate RCy-PDI_2_ molecules with intramolecularly trapped toluene between the PDI units (Figure S12). The centroid-to-centroid distance (*d*) between the intramolecular PDI units of the RCy-PDI_2_ aggregate was *d* ≈ 7.0 Å, and was moderately larger for the toluene-trapped structure (*d* ≈ 7.2 Å). Notably, the resolved 3D ED structures were unable to resolve the precise location of the toluene molecules in the aggregate and hence seven possible structures were proposed based on the electron density distribution of the toluene. The combined stack of possible structures obtained from 3D ED measurements indicated selective diffusion of toluene molecules along the *c*-axis when viewed from the *a*-axis (Figure 5c). The orientation of the trapped toluene molecules in the RCy-PDI_2_ aggregate stack has the toluene molecular axis tilted by ∼90° relative to the PDI molecular axis (Figure S11and S15). The relative spacing within the stack of the RCy-PDI_2_ aggregate may allow toluene to reorient and diffuse anisotropically through the crystal. The results indicate that the phase propagation in the RCy-PDI_2_ aggregate could arise from the combined contributions of anisotropic diffusion^35,36^ and detrapping.^37^ Although local crystal domains, intrinsic packing defects, or polymorphism^38^ can influence the optical response of molecular crystals, the spatial propagation of the phase boundary observed by time-dependent FLIM, together with the single crystal structure obtained from 3D ED and the evidence for lattice-trapped toluene, supports a dynamic solvent-mediated structural relaxation mechanism rather than static structural heterogeneity.

To verify the crystallite stability upon toluene detrapping, time-dependent hot air drying, followed by powder X-ray diffraction (PXRD) measurements of the RCy-PDI_2_ aggregates at different drying stages (Figure 5d). Simulated PXRD data of the RCy-PDI_2_ aggregates with the detrapped toluene was generated to trace the trend in the PXRD peaks with toluene evaporation. Upon comparison with the simulated PXRD data of the RCy-PDI_2_ aggregate with masked toluene, drying preserves crystallinity, and the RCy-PDI_2_ aggregate dried for more than an hour maintains a PXRD pattern similar to the simulated spectrum. Peaks III and VI depict significant intensity in the simulated PXRD spectrum but are not observed in the experimental PXRD measurements. The moderate/local disorder induced in the crystalline structure by solvent detrapping is likely responsible for the weak signal intensity observed in the experimental PXRD measurement.^39^ To further understand the packing, solid-state NMR measurements at 60 kHz spinning speed were performed on RCy-PDI_2_ aggregate, as shown in Figures S16–S18.

A suite of solid-state NMR measurements was performed to understand the local structural information about the protons and carbons in the RCy-PDI_2_. ^1^H–^13^C Heteronuclear correlation (HETCOR) measurements were performed on the RCy-PDI_2_ aggregate to examine the correlation between aliphatic and aromatic moieties (Figure S16). In the ^1^H–^1^H double quantum-single quantum correlation NMR spectrum, a correlation between aliphatic and aromatic protons was observed for the RCy-PDI_2_ aggregate (Figure S17). Furthermore, the ^13^C cross-polarization measurements of the RCy-PDI_2_ aggregate show a correlation between the protons on the aliphatic and aromatic regions (Figure S18). Solid-state NMR chemical shift assignments for the RCy-PDI_2_ aggregate, complemented by solution-state NMR data (Figures S19 and S20), were determined and compared directly with CASTEP-calculated chemical shifts. The root-mean-square deviation (RMSD) value between the CASTEP calculations and the experimental solid-state NMR chemical shifts validates the accuracy of the RCy-PDI_2_ aggregate structure (RMSD = 3.76 ppm).^40,41^ NCI plot of the toluene-trapped structure reveal an extended region of weak attractive interaction (reduced density gradient isosurface, s = 0.5 au, green isosurface) spanning the face of the toluene methyl group and the PDI bay area, consistent with CH−π and dispersive interactions stabilizing the guest.

To quantify the excitonic consequences of toluene intercalation, we performed Coulombic coupling calculations (EET) on the crystallographically resolved toluene-trapped geometry and a toluene-free model obtained from the 3D ED structure with the guest molecule removed (Figures S21 and S22). The EET calculations reveal that the toluene-trapped RCy-PDI_2_ structure exhibits a strong intermolecular J-type Coulombic coupling [J_c_ = -55 to -73 meV], consistent with the red-shifted absorption diagnostic of J-type aggregate behavior.^42^ The range of Coulombic coupling in the RCy-PDI_2_ structure is defined by two energetically proximal states that can couple to contribute to the overall excitonic coupling (Figure S21). Critically, this coupling arises not from a cofacial, excimer-prone geometry, but from the splayed, V-shaped conformation (interplanar angle: 26.3°) enforced by the intercalated toluene. The intercalated molecular structure separates the intramolecular PDI centroids by ∼7.2 Å and aligns the transition dipoles in a head-to-tail arrangement favorable for constructive excitonic interference (Figure 5c). In the toluene-free structure, the relaxation of steric constraints imposed by the guest molecule permits optimal intermolecular interactions between neighboring RCy-PDI_2_ molecules in the stack (intermolecular distance: ∼3.5 Å). This geometric shift promotes short-range orbital overlap between adjacent chromophoric units, which is the hallmark of excimer formation, consistent with the long-lived emission (τ_green_ ≈ 8 ns) and red-shifted spectral profile (∼650 nm) observed in the toluene-depleted domains of the FLIM image. Despite emissive charge-transfer interactions between electron-deficient aromatic chromophores and aromatic solvents have been reported for NDI derivatives,^43^ our observations are more consistent with a PDI–PDI excimer.^22,44^ The long-lived emission emerges upon solvent detrapping rather than in the presence of trapped toluene, and the crystallographic analysis indicates that the primary role of toluene is to modulate the intermolecular packing and excitonic coupling rather than to serve as an emissive charge-transfer counterpart. Overall, the Coulombic coupling calculations reveal that toluene acts as a geometric spacer that enforces the precise intermolecular geometry required for long-range Coulombic coupling, and its removal collapses this geometry into a short-range orbital-overlap regime.

To examine the generality of the observed phase propagation behavior, we further investigated the aggregation of RCy-PDI_2_ in two aromatic solvents, namely, benzene (Bp: 80 °C) and *o*-xylene (Bp: 144 °C). Time-resolved FLIM measurements revealed two discrete fluorescence lifetime populations in aggregates formed from both solvents, indicating the coexistence of distinct emissive domains analogous to those observed in toluene (Figure S23). In benzene, RCy-PDI_2_ self-assembled into fibrillar aggregates closely resembling those obtained from toluene, exhibiting short- and long-lifetime domains centered at approximately 2 and 11 ns, respectively. Time-dependent FLIM imaging revealed a rapid evolution of the lifetime distribution, with the long-lifetime population accounting for ∼80% of the FLIM image within 180 min (from 62%), indicative of accelerated phase propagation relative to the toluene-derived aggregates (Figure S23a and S23b). In contrast, aggregation from *o*-xylene produced predominantly spherulitic morphologies with comparatively shorter longitudinal dimensions. Although these aggregates likewise exhibited two discrete fluorescence lifetime populations (∼2 ns and ∼10.5 ns), their temporal evolution was markedly slower. Even after 270 min, only a minute change (∼1%) in the lifetime population profile was observed, indicating substantially suppressed phase propagation (Figure S23c and S23d). We attribute this pronounced solvent dependence to differences in solvent mobility within the aggregate (Figure S23e and 23f), where the larger molecular size and higher boiling point of *o*-xylene likely retard solvent detrapping and diffusion, thereby slowing the progression of the phase boundary. Collectively, these observations demonstrate that while excitonic heterogeneity is retained across different aromatic solvents, the kinetics of phase propagation are strongly governed by solvent-dependent diffusion dynamics and solvent–aggregate interactions.

Taken together, we have elucidated the phase propagation phenomena in the chiral perylenediimide dimer aggregate fibrils. The detailed FLIM and spectroscopic analysis elucidated the excitonic nature of the solid-to-solid phase transformation observed in the aggregate fibrils. 3D ED measurements of RCy-PDI_2_ revealed the aggregate structure dictating the excitonic switching with toluene detrapping and the possibility of anisotropic diffusion of toluene along a particular orientation. The peculiar aggregate stacking has facilitated optical and diffusion anisotropies, thereby dictating phase propagation in the molecular aggregate fibril.

## Conclusions

In conclusion, we report the first example of solid-to-solid phase transformation via phase propagation in a molecular aggregate fibril. The time-dependent FLIM measurements revealed a phase transformation from a short lifetime species to a long lifetime species in the observed RCy-PDI_2_ aggregate fibrils. The FLIM and emission measurements indicated the possibility of J-type aggregate and excimer formation for the blue and green phases, respectively. The nature of the RCy-PDI_2_ aggregates was further confirmed by steady-state measurements and supported by excitonic coupling and TDDFT calculations. 3D ED measurements revealed the RCy-PDI_2_ aggregate structure with the toluene intramolecularly trapped between the PDI units. The controlled cooling and drying experiments further reinforced the important role of toluene in the excitonic switching in the RCy-PDI_2_ aggregate fibrils. The possible structures obtained from the 3D ED measurements indicated anisotropic diffusion of toluene along the *c*-axis when viewed along the *a*-axis. Overall, the anisotropic diffusion and detrapping of toluene contributed to the phase propagation and the structural relaxation from toluene detrapping resulted in the excitonic switching from J-type aggregate to excimer formation. The work highlights the importance of understanding phase propagation and evolution of structural heterogeneity in the development of advanced optoelectronic and functional materials.

## Supplementary Material



## Notes

Synthesis and characterization, experimental methods, tables with crystallographic parameters and CASTEP analysis, NMR experiments (^1^H NMR, ^13^C DEPT 135, ^1^H–^1^H COSY, ^1^H–^13^C HETCOR, etc.), FLIM and spectrograph measurements, morphological characterization (TEM, SEM, microscope images, etc.), crystal structure analysis, computational analysis (NCI analysis and EET coupling calculations) (PDF)

## Abbreviations

3D ED: Three-dimensional Electron Diffraction
FLIM: Fluorescence Lifetime Imaging Microscopy
NMR: Nuclear Magnetic Resonance
RCy-PDI_2_: (*1R,2R*) Cyclohexadiamine-based perylenediimide dimer
TOL: Toluene
fwhm: Full Width at Half Maximum
CPL: Circularly Polarized Luminescence
SPAD: Single Photon Avalanche Diode
SEM: Scanning Electron Microscopy
TEM: Transmission Electron Microscopy
PXRD: Powder X-ray Diffraction
HETCOR: Heteronuclear Correlation
CASTEP: Cambridge Sequential Total Energy Package
RMSD: Root Mean Square Deviation
