# Supplementary material for: Solvent-Mediated Phase Propagation Governs Excitonic Heterogeneity in Chiral Fibrils
Source: ACS Cent Sci. 2026 Aug 28;12(9):1468–76. doi: 10.1021/acscentsci.6c00969 (PMC13614061; doi:10.1021/acscentsci.6c00969)
Supplement: Supplementary file 1 [file oc6c00969_si_001.pdf]

Solvent-Mediated Phase Propagation Governs Excitonic Heterogeneity in Chiral Fibrils

Philip Daniel Maret, [a] Brijith Thomas*[b] and Mahesh Hariharan*[a]

[a]School of Chemistry, Indian Institute of Science Education and Research Thiruvananthapuram (IISER TVM), Maruthamala P.O., Vithura, Thiruvananthapuram 695551, Kerala, India.

*E-mail: mahesh@iisertvm.ac.in

[b]Science Division, New York University Abu Dhabi, P.O. Box 129188, Abu Dhabi, United Arab Emirates.

*E-mail: brijiththomas@nyu.edu

Supporting Information (SI)

Table of Contents

Section 1: Syntheses and Characterization SI 4

Section 2: Experimental Methods SI 5

Section 3: Tables

Table S1: Comparison table highlighting the significance of the present work in the field of photophysics and supramolecular chemistry……………………………………… SI 8

Table S2: Representative spectral shifts of the ribbon fibers with blue and green regions in the diverse FLIM images……………………………………………………… SI 8

Table S3: 3D ED data collection details (room temperature) for RCy-PDI$_2$ aggregates used in refinement………………………………………………………………………...... SI 8

Table S4:3D ED data collection details (cryo) for RCy-PDI$_2$ aggregate used in refinement………………………………………………………………………………… SI 8

Table S5: The crystallographic details of 3D ED kinematic refinement against merged data from the crystals……………………………………………………………. SI 9

Table S6: The carbon numbering for the RCy-PDI_2 molecule is given below. Comparison of the solution and solid-state NMR chemical shift along with the difference between the chemical shifts is tabulated………………………………………….. SI 10

Section 4: Figures

Figure S1: Time-dependent FLIM measurements of the Cy-PDI_2 aggregate fibrils, monitored from 0 to 180 minutes. The FLIM images clearly depict the phase propagation in the aggregate fibrils and the fluorescence lifetime distribution clearly indicates the transformation from blue phase to green phase…………………………… SI 12

Figure S2: Representative FLIM images of the aggregates of RCy-PDI_2 in toluene drop-casted on a coverslip surface……….................…….. SI 12

Figure S3: Representative FLIM images of ribbon-like aggregates of RCy-PDI_2 formed in TOL (a, c) and the corresponding emission spectra recorded from the spectrograph (b, d). The blue and green circles with plus show the regions of interest within the aggregates from which the respective emission spectrum was obtained...….. SI 13

Figure S4: a) Concentration-dependent steady-state absorption and b) fluorescence emission measurements of RCy-PDI_2 in toluene..………………………….................... SI 13

Figure S5: SEM images of the RCy-PDI_2 aggregates in TOL showing diverse fibrils, ribbon-like structures, and some complex secondary structures formed from fibrils…………………… SI 14

Figure S6: Time-dependent evolution of the aggregates of RCy-PDI_2 in toluene monitored through SEM imaging technique……………………………………………..... SI 14

Figure S7: TEM images of the aggregates show that small fibril-like structures are formed, which seed the growth of bigger ribbon-like structures…………………………... SI 15

Figure S8: a) - e) The TEM diffraction pattern and f) - i) TEM images of the aggregate superstructures along with the scale. The TEM measurements were carried out to determine the crystallinity for the 3D ED measurements. ……………………............... SI 15

Figure S9: The confocal microscopy images of RCy-PDI_2 aggregate fibres with 100x magnification showing distinct bands upon white light excitation………………………… SI 16

Figure S10: Polarisation microscopy images of the RCy-PDI_2 aggregate fibrils with a) perpendicular and b) parallel orientation of the polariser..………………………………… SI 16

Figure S11: The supernatant solution was boiled at 140 °C until all the particles of the molecules were completely dissolved. The RCy-PDI_2 solution was systematically cooled with 10 °C interval until 60 °C. Small aggregate fibrils observable to naked eyes starts to develop in the aggregate solution. The RCy-PDI_2 aggregate solution is cooled to room temperature and drop-casted freshly for the FLIM measurements……………... SI 17

Figure S12: The crystal structure for the RCy-PDI_2 crystalline aggregate obtained from 3D ED shown along a) a-axis, b) b-axis and c) c-axis..…………………………………... SI 17

Figure S13: The interplanar angle between the two PDI units of the RCy-PDI_2 solved SI 18

structure. The angle is measured at the intersection of the two planes…………………

Figure S14: NCI analysis of the RCy-PDI$_2$ aggregate structure obtained from the 3D ED measurement. The prominent π-π interaction between the PDI units intermolecularly and the toluene-stacked PDI units also has an enhanced π-π interaction attributed to the J-type coupling in the aggregate………………………………… SI 18

Figure S15: The representative packing of the aggregate in a) along a a-axis, which gives the perspective of alternating orientation of the RCy-PDI$_2$ molecules, b) along the b-axis, which gives the view of π-π stacking in the RCy-PDI$_2$ molecules, and c) along the c-axis of the crystalline fibre which shows the prominent interaction of the intermolecular PDI units responsible for the excimer formation………………………….. SI 19

Figure S16: ^1H-^{13}C HETCOR spectra of the RCy-PDI$_2$ aggregate in solid state collected at long contact time…………………………………………………………………….. SI 19

Figure S17: The ^1H-^1H BABA spectrum of the RCy-PDI$_2$ aggregate in solid state collected at 60 kHz spinning speed. The arrows represent the correlation peaks between the aliphatic and aromatic peaks in the 2D spectrum.. ………………………….. SI 20

Figure S18: The ^1H NMR spectra of the RCyPDI$_2$ collected at 60 kHz, along with the 1D ^{13}C CP solid state NMR spectra…………………………………………………………... SI 21

Figure S19: ^{13}C DEPT 135 spectrum of the RCy-PDI$_2$ molecule in $CDCl_3$……………….. SI 22

Figure S20: The ^1H-^1H COSY spectrum of the RCy-PDI$_2$ molecule in $CDCl_3$…………… SI 22

Figure S21: The schematic representation of the energy profiles calculated using TDDFT calculations in Gaussian 16.1 for the RCy-PDI$_2$ molecule, where the Dimer structure coordinates are considered from the crystal structure and the Dimer intercalated with a toluene molecule is also evaluated and compared with the unit cell structure (Dimer and Dimer with toluene). The lower energy bright state of the aggregate unit confirms the J-type coupling in RCy-PDI$_2$ aggregate. EET calculations show strong J-type coupling between the two bright states and energetically proximal states of the dimer fragments considered for the calculation. SI 23

Figure S22: The Gaussian fragments employed for the EET calculations of the aggregate. The toluene intercalated structure is fragment 1(red highlight) and the toluene detrapped dimer is assigned as fragment 2 (green highlight). SI 23

Figure S23: The time-dependent FLIM measurements of RCy-PDI$_2$ aggregates in benzene from a) 0 min to b) 180 min. Time-dependent FLIM measurements of the RCy-PDI$_2$ aggregates in o-xylene at c) 0 min and b) 270 min. The fluorescence lifetime population of the long and short fluorescence lifetime components of the FLIM acquired at different time frames of the aggregates formed in e) benzene and f) o-xylene. SI 24

Reference SI 25

Section 1: Syntheses and Characterization

The compounds were synthesized according to the previously reported procedure.[1]

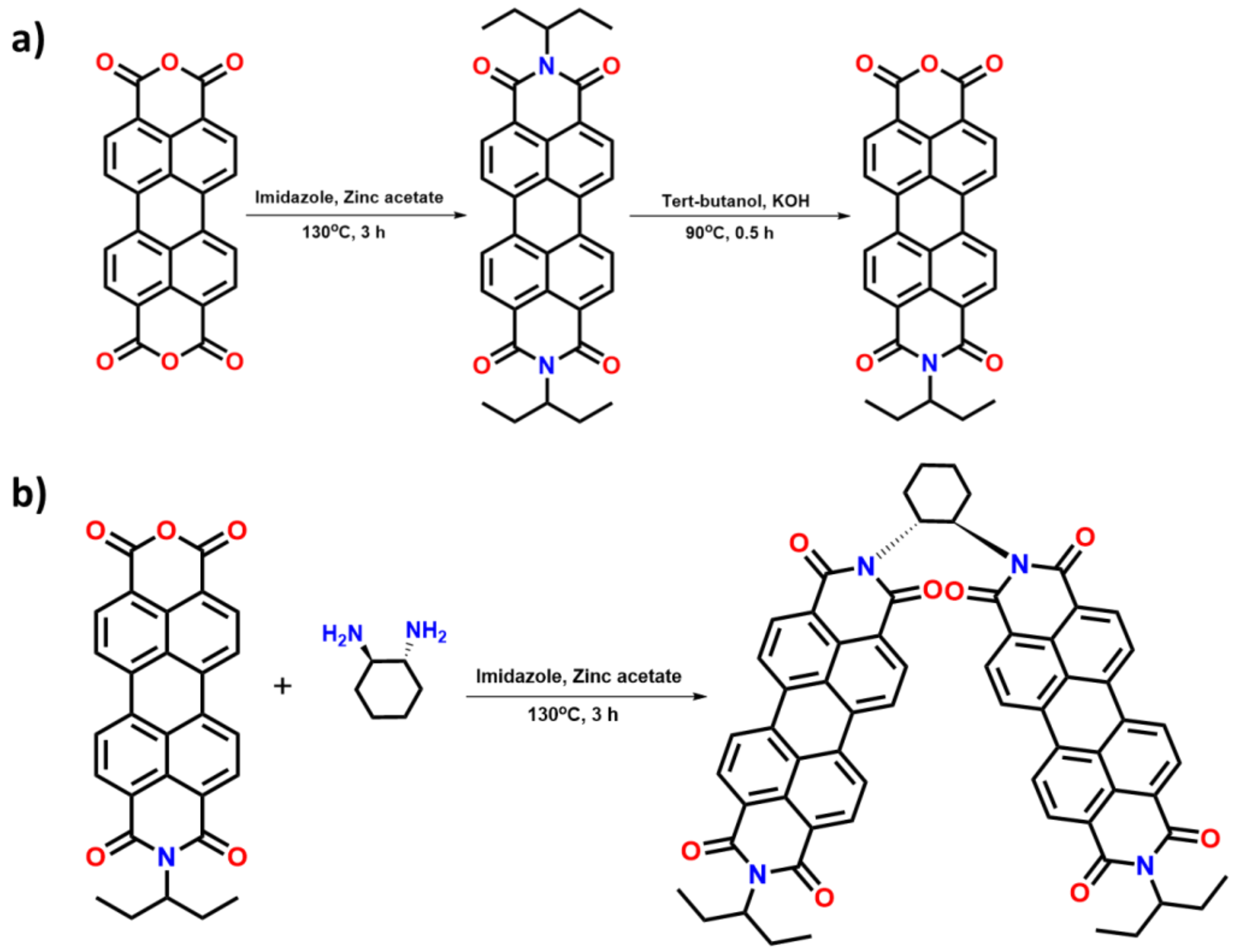

Scheme S1: a) Synthesis scheme for the perylenemonoimide monoanhydride. b) Synthesis scheme for RCy-PDI$_2$ as per the reported procedure.

Section 2: Experimental Methods

Materials and Methods

All chemicals were procured from commercial suppliers and utilized as received without further purification. All reactions were carried out in oven-dried glassware prior to use. Solvents were dried and distilled by standard laboratory purification techniques. Thin-layer chromatography (TLC) analyses were performed on recoated aluminium plates of silica gel 60 F254 plates (0.25 mm, Merck). TLC plates were visualized under short- and long-wavelength UV lamps. Column chromatography was performed using silica gel of 200-400 mesh employing a solvent polarity correlated with the TLC mobility observed for the substance of interest. Photophysical measurements of the aggregates were performed in a 10 mm path-length cuvette. Absorption and emission spectra were recorded on Shimadzu UV-3600 UV-VIS-NIR and Horiba Jobin Yvon Fluorolog spectrometers, respectively.

Computational Analysis

All the calculations were carried out in Gaussian 16.1.[2] Vertical excitation energies and oscillator strengths were calculated employing time-dependent DFT (TDDFT) at the CAM-B3LYP-D3/def2svp level of theory. In order to compute long-range Coulombic coupling, an electronic energy transfer (EET) module available in Gaussian 16.1 was utilized.

Fluorescence Lifetime Imaging Measurements

Fluorescence lifetime imaging microscopy (FLIM) images were acquired using the MicroTime 200 (MT200) time-resolved fluorescence microscope by PicoQuant. The inverted microscope (Olympus IX83) was equipped with a piezo scanning stage (P-733.2CD, PI). Sample excitation was achieved using a 512 nm pulsed laser (LDH-D-C-512, PicoQuant) with a repetition rate of 20 MHz, and scanning was performed with a dwell time of 0.4 ms and 0.6 ms. A water-immersion objective (UplanSApo 60×, NA 1.2) collected the photons. Spectral filtering utilized a major dichroic mirror (zt405-442/510 rpc-UF3, Chroma) and a 519 nm long-pass emission filter (FF01-519/LP, Semrock). A pinhole (50 μm) spatially filtered the photons, which were then directed to a single-photon avalanche photodiode (SPAD) detector (Excelitas SPAD). The detector signals were postprocessed using the HydraHarp 400 time-correlated single-photon counting (TCSPC) data acquisition unit (PicoQuant). SymPhoTime64 software (PicoQuant) handled data recording and analysis. The excitation laser operated at a repetition rate of 20 MHz with a power of ~0.12 μW. An Andor spectrograph (Kymera 193i) from Oxford Instruments was employed to record the fluorescence emission from different segments of the aggregate fibril.

Preparation and Photophysical Measurements of Aggregates

The samples for the aggregates were prepared by weighing 1 mg of RCy-PDI_2 into 1 mL of toluene and heating to 130 °C until completely dissolved, yielding a transparent solution. Small fibre-like structures are visible to the naked eye in the solution after one hour. The aggregate solution was drop-casted on a glass coverslip for the solid-state absorption and emission experiments. Aggregates prepared at different concentrations were used for concentration-dependent steady-state absorption, fluorescence emission, circular dichroism, and circularly polarised luminescence measurements.

3D Electron Diffraction

Samples were deposited on standard TEM grids (amorphous carbon on Cu) as dry powder without grinding and measured on an ELDICO ED-1 electron diffractometer using the software ELDIX.[3] The device is equipped with a LaB6 electron source operating at an acceleration voltage of 160 kV (λ = 0.02851 Å) and a hybrid-pixel detector (Dectris QUADRO). Measurements were performed both at room temperature and 100 K. For the cryo measurement, the sample was plunged into liquid nitrogen and transferred cold and protected from air to the diffractometer. The grid was screened for suitable crystals in STEM (scanning transmission electron microscopy) mode and diffraction data were recorded in continuous rotation mode with a pseudo-parallel beam of ca. 750 nm diameter. Parts of the measurements affected by beam damage or shadowing by the grid were omitted. Measurement details for all datasets used are given in Tables S2 and S3.
Data were processed using the APEX4 software package.[4] Frames were integrated separately for each crystal, then merged, scaled, and corrected for Lorentz effects, scan speed, background, and absorption using SAINT and SADABS.[5,6] Space group assignment was based on systematic absences, E statistics, and successful refinement of the structure. The structure was solved using ShelXD and refined with ShelXL in conjunction with ShelXle and Olex2.[7–10] Least squares refinements were carried out within the kinematic approximation by minimizing Σw(Fobs2-Fcalc2)2 with the ShelXL weighting scheme and using neutral electron scattering factors.[8,11] Non-H atoms were refined with anisotropic displacement parameters. Restraints on geometries and APDs were used to ensure convergence within physically meaningful limits. H atoms were placed in calculated positions based on typical distances for neutron diffraction and refined using riding model with U_{iso}(H) = 1.5·Ueq(C) for methyl and U_{iso}(H) = 1.2·Ueq(C) for other groups.

Deposition Number 2493726-2493727 contains the supplementary crystallographic data for this paper. These data are provided free of charge by the joint Cambridge Crystallographic Data Centre and Fachinformationszentrum Karlsruhe Access Structures service and can be accessed at www.ccdc.cam.ac.uk/structures.

Circular Dichroism and Circularly Polarised Luminescence Measurements

Circular dichroism (CD) spectra were recorded using a JASCO J-815 spectropolarimeter equipped with a Peltier-controlled thermostatted cell holder, employing a quartz cuvette with a 1 mm optical path length. Circularly polarized luminescence (CPL) measurements were carried out on a JASCO CPL-300 spectrophotometer featuring double-prism excitation and emission monochromators to minimize stray light and eliminate spurious linear polarization effects, using a quartz cuvette with a 1 mm path length.

Powder X-ray Diffraction (PXRD) Measurements and Simulation

PXRD measurements for the RCy-PDI_2 aggregate drying experiments were carried out using the Bruker D8 Advance PXRD diffractometer. The simulated PXRD measurement and the corresponding planes were obtained from the Avogadro software.

Solid-state NMR Measurements

All solid-state NMR measurements were collected at 60 kHz spinning speed on a Bruker Avance HD 600 WB NMR spectrometer. Finely powdered samples were filled in 1.3 mm rotor and spun at 60 kHz. The ^{1}H and ^{13}C chemical shifts are calibrated relative to neat tetramethylsilane (TMS) using adamantane as a secondary chemical shift standard. Single-pulse ^{1}H spectra were recorded using 90° rf pulses operating at the ^{1}H nutation frequency ν_{H} ≈ 185 kHz, 16 accumulated NMR-signal transients, and relaxation delays (τ_{relax}) of 2.0 s. The ^{13}C spectra were recorded by the $^{1}H \rightarrow {}^{13}C$ CP at the double quantum Hartmann−Hahn condition, $\nu_{H} + \nu_{C} = \nu_{rf,}$ which involved ramped CP of ν_{H} = 20 ± 5 kHz for ^{1}H (vC = 40 kHz), a 1.5 μs 90° ^{1}H pulse, and spinal-64 ^{1}H decoupling at v_{H} = 150 kHz. The $^{13}C\{^{1}H\}$ 2D HETCOR NMR spectra were recorded with τ_{CP} = 1.0 ms, τ_{relax} = 2.0 s, and dwell times of Δt_2 = 20.0 μs and $\Delta t_1 = 5\tau_r$ = 83.34 μs, where $\tau_r = \nu_{r-1}$ is the rotor period. 32(t_1) × 2048(t_2) time points were collected with 2048 accumulated transients per t_1 value. The 2D NMR data sets were zero-filled to 64 t_1 points, along with 4096 (t_2), and were apodized by a cos2θ and an exponential function along the indirect and direct dimensions, respectively, with the latter giving a 50 Hz FWHM Lorentzian broadening.

CASTEP Calculations

Plane-wave DFT calculations were done with the gauge-including projected augmented wave (GIPAW) approach as implemented in the CASTEP version 2017.[12,13] Generalised gradient approximation with the exchange-correlation PBESOL functional was used for optimising the geometry and calculating the NMR properties.[14] The Tkatchenko and Scheffler method was used for the dispersion corrections.[15] The calculation efficiency was maximized with an energy cut off of 650 eV with a Monkhorst-Pack grid with a k-point spacing of 0.07 $Å^{-1}$.[16]

Coulombic Coupling Calculation

Electronic energy transfer (EET), also referred to as resonance energy transfer (RET), describes the non-radiative transfer of excitation energy from a photoexcited donor to a proximal acceptor in its ground state. In *Gaussian 16.1*, EET is treated within a quantum mechanical framework based on a DFT description of the electronic wavefunction, combined with a time-dependent variational formalism. This approach involves performing excited-state calculations for each fragment independently, followed by evaluation of the electronic couplings between all resulting excited states. For singlet excitation energy transfer (SEET), the total electronic coupling is composed of three distinct contributions:

$$V^{SEET} = V^{Coul} + V^{exch} + V^{ovlp} \qquad (1)$$

Where, V^{Coul} is the Coulombic coupling between electronic transitions, V^{exch} is the Dexter's exchange coupling and V^{ovlp} is the term arising from the overlap of donor-acceptor orbitals.

$$V^{Coul} = \iint dr_1 dr_2 \frac{\rho_D^{tr*}(r_1)\rho_A^{tr}(r_1)}{|r_1 - r_2|} \qquad (2)$$

Where, $\rho_D^{tr*}(r_1)$ and $\rho_A^{tr}(r_1)$ are the transition densities (the diagonal element of the density matrix), respectively, of the donor and acceptor.

TDDFT calculation was performed on RCy-PDI_2 aggregate after defining each RCy-PDI_2 as a fragment for the S0 to S1 transition at CAM-B3LYP D3/DEF2-TZVP, and Coulombic coupling between the states was procured.[17–19]

Section 3: Tables

Table S1: Comparison table highlighting the significance of the present work in the field of photophysics and supramolecular chemistry.

Reference	Method	Molecular System	Key Findings
Hariharan, Thomas and Maret	FLIM, 3D-ED, TEM, and Solid-state NMR	Chiral perylenediimide dimer aggregate fibers	Phase propagation mediated excitonic switching
Würthner, Kim and co-workers (*Nat. Chem.*, ***2025***, 17, 767–776)	2D-NMR and geometry-optimized structures	Perylenediimide multimers	Photoinduced stepwise charge hopping
Thomas, Hariharan, Brazda and co-workers (Chem. Sci., ***2024***, 15, 490-499)	TEM, 3D-ED and Solid-state NMR	Nitroperylenediimide	Exploring packing in partial disordered structure
Carem and co-workers (*Nat. Chem.*, ***2024***, 16, 800–808)	Cryo-EM	Amphiphilic cyanine dye	Excitonic coupling in a light-harvesting J-aggregate nanotubes
Jeirry, Schumutz and co-workers (*ACS Nano* **2024**, 18, 44, 30448–30462)	3D Cryo-EM	Fmoc-FFY tripeptide	Determination of the structure of a nanofiber
Würthner, Kim and co-workers (J. Am. Chem. Soc., ***2018***, 140, 4253–4258)	Geometry optimized structures	Perylendiimide dimers and oligomers	Exciton migration and excimer formation
Lehmann, Würthner and co-workers (*Nat Commun.*, ***2018***, **9**, 2646)	MAXS, WAXS, PXRD	Perylenediimides	Liquid crystalline J-aggregates
Schröder, Willbold and co-workers (*Science*, ***2017***, 358, 116-119)	Cryo–electron microscopy	Amyloid-β(1–42)	Structural determination of the amyloid-β(1–42)
Griffin and co-workers (*J. Am. Chem. Soc.*, ***2016***, 138, 30, 9663–9674)	MAS-NMR and STEM	Aβ42	Atomic resolution structure of monomorphic $A\beta_{42}$ amyloid fibrils.

Table S2: Representative spectral shifts of the RCy-PDI_2 fibers with blue and green regions in the diverse FLIM images.

Aggregate Fibers	Emission Maxima (nm)		Spectral Shift (nm)
	Blue	Green	Δλ
**1**	600	640	40
**2**	605	649	44
**3**	607	649	42
**4**	603	644	41
**5**	600	641	41

Table S3: 3D ED data collection details (room temperature) for RCy-PDI_2 aggregates used in refinement.

Crystal no.	Approximate size [µm]	Angular range [°]	Rotation per frame [°]	Exposure time [s]	Total exposure [s]	Frames measured	Frames used
1	2.5 x 0.9 x 0.5	−50 to +50	0.5	0.5	100	200	140
2	7.5 x 0.9 x 0.4	−40 to +60	0.5	0.5	100	200	140
3	3.0 x 0.6 x 0.6	−50 to +50	0.5	0.5	100	200	140
4	2.8 x 1.1 x 0.9	−60 to +60	0.5	0.5	120	240	130
5	1.6 x 0.8 x 0.6	−60 to +60	0.5	0.5	120	240	150
6	4.8 x 0.8 x 0.6	−40 to +60	0.5	0.5	100	200	140
7	2.5 x 0.9 x 0.6	−50 to +60	0.5	0.5	110	220	110
8	>9 x 2.2 x 0.8	−60 to +60	0.5	0.5	120	240	120
9	4.5 x 1.0 x 0.8	−40 to +60	0.5	0.5	100	200	130

Table S4: 3D ED data collection details (cryo) for RCy-PDI_2 aggregate used in refinement.

Crystal no.	Approximate size [µm]	Angular range [°]	Rotation per frame [°]	Exposure time [s]	Total exposure [s]	Frames measured	Frames used
1	4.9 x 0.9 x 0.5	−60 to +60	0.5	0.5	120	240	100
2	1.6 x 1.0 x 0.4	−60 to +60	0.5	0.5	120	240	160
3	2.6 x 0.9 x 0.7	−60 to +50	0.5	0.5	110	220	160
4	3.1 x 0.8 x 0.7	−60 to +40	0.5	0.5	100	200	160
5	3.5 x 1.0 x 0.8	−50 to +60	0.5	0.5	110	220	130
6	1.4 x 0.7 x 0.4	−30 to +40	0.5	0.5	70	140	110
7	4.2 x 0.6 x 0.5	−60 to +60	0.5	0.5	120	240	130
8	2.7 x 1.0 x 0.8	−50 to +60	0.5	0.5	110	220	180
9	4.5 x 0.8 x 0.5	−40 to +40	0.5	0.5	80	160	90

Table S5: The crystallographic details of 3D ED kinematic refinement against merged data from the crystals.

Parameters	Values
a, b, c (Å)	15.34(8), 18.29(9), 21.29(11)
α, β, γ (°)	90.00, 96.34, 90.00
Space group	$P2_1$
Formula	$C_{64}H_{48}N_4O_8$
Independent reflections	8061
Parameters	1370
Restraints	3570
Resolution (Å)	1.10
Completeness (%)	86.6
R_{int} (%)	21.64
$R_1[1>2\sigma(I)]$ (%)	14.65
wR_2 [all data] (%)	36.43
Goodness of fit	1.04

Table S6: The carbon numbering for the RCy-PDI$_2$ molecule is given below. Comparison of the solution and solid-state NMR chemical shifts along with the difference between the chemical shifts is tabulated.

IUPAC	Chemical Shift (soln)	Chemical Shift (solid)	Predicted chemical shift	Difference
1	131.42	132.95	128.19	4.761825
2	131.38	129.07	132.05	-2.98297
3	125.13	124	122.06	1.944875
4	125.13	124	121.13	2.8697
5	131.38	129.07	128.35	0.71633
6	131.42	132.95	126.15	6.79644
7	131.1	131.86	127.53	4.333805
8	131.08	130	132.44	-2.44237
9	128.34	128	122.47	5.52627
10	128.34	128	122.45	5.54574
11	131.08	130	131.42	-1.420195
12	131.1	131.86	125.79	6.066635
13	122.66	122	126.96	-4.961565
14	122.55	122	123.71	-1.710075
15	122.5	122	123.95	-1.95345
16	128.34	122	128.85	-6.850155
17	128.27	128	131.71	-3.712245
18	128.27	128	133.44	-5.43534
19	130.28	130	133.66	-3.659245
20	130.58	130	132.51	-2.510515
21	162.63	162.1	160.39	1.708445
22	162.63	163.94	160.11	3.83076
23	162.63	161.8	164.33	-2.53423
24	162.63	163.93	161.38	2.545475
25	53.11	56.99	57.95	-0.96015
26	24.7	25.84	25.36	0.48263
27	11.1	10.3	13.65	-3.346165
28	24.7	25.84	27.04	-1.201525
29	11.1	11.65	9.23	2.423525
1'	57.42	58.37	54.75	3.622665
2'	29.05	25.73	31.71	-5.984325
3'	25.4	26	25.80	0.204555
4'	25.4	26	27.17	-1.16808
5'	29.05	25.73	29.74	-4.00812
6'	57.42	58.37	54.19	4.17756
22a	162.63	163.94	160.57	3.373215
4a	125.13	125	121.63	3.373215
5a	131.38	129.07	129.27	-0.19876
6a	131.42	132.95	126.39	6.5628
19a	130.28	130	133.83	-3.834475
20a	130.58	130	132.41	-2.413165
7a	131.1	131.86	127.72	4.139105
8a	131.08	130	132.29	-2.28661
9a	128.34	128	122.06	5.944875
24a	162.63	163.93	161.52	2.409185
23a	162.63	161.8	164.71	-2.913895
10a	128.34	128	122.49	5.5068
11a	131.08	130	132.16	-2.160055
12a	131.1	131.86	127.28	4.57718
15a	128.27	128	133.25	-5.250375
17a	128.27	128	132.41	-4.413165
19a	125.08	125	126.08	-1.07568

2a	131.38	129.07	131.32	-2.252845
3a	125.13	125	121.28	3.723675
21a	162.63	162.1	160.66	1.435865
13a	122.66	122	127.48	-5.47752
14a	122.55	122	123.75	-1.749015
15a	122.5	122	124.02	-2.021595
16a	128.34	122	128.34	-6.343935
25a	53.11	56.99	56.99	0.003615
26a	24.7	25.84	28.22	-2.37946
27a	11.1	10.3	9.68	0.61598
28a	24.7	25.84	25.16	0.67733
29a	11.1	11.65	11.45	0.203945
1a	131.42	132.95	126.08	6.87432
18a	128.27	128	133.25	-5.250375
**RMSD**				3.76 ppm

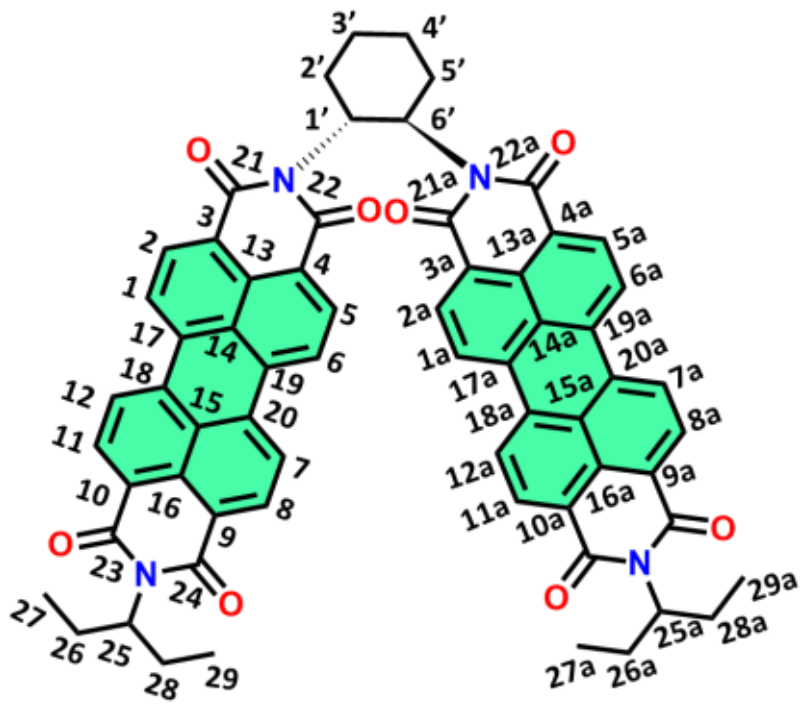

Section 4: Figures

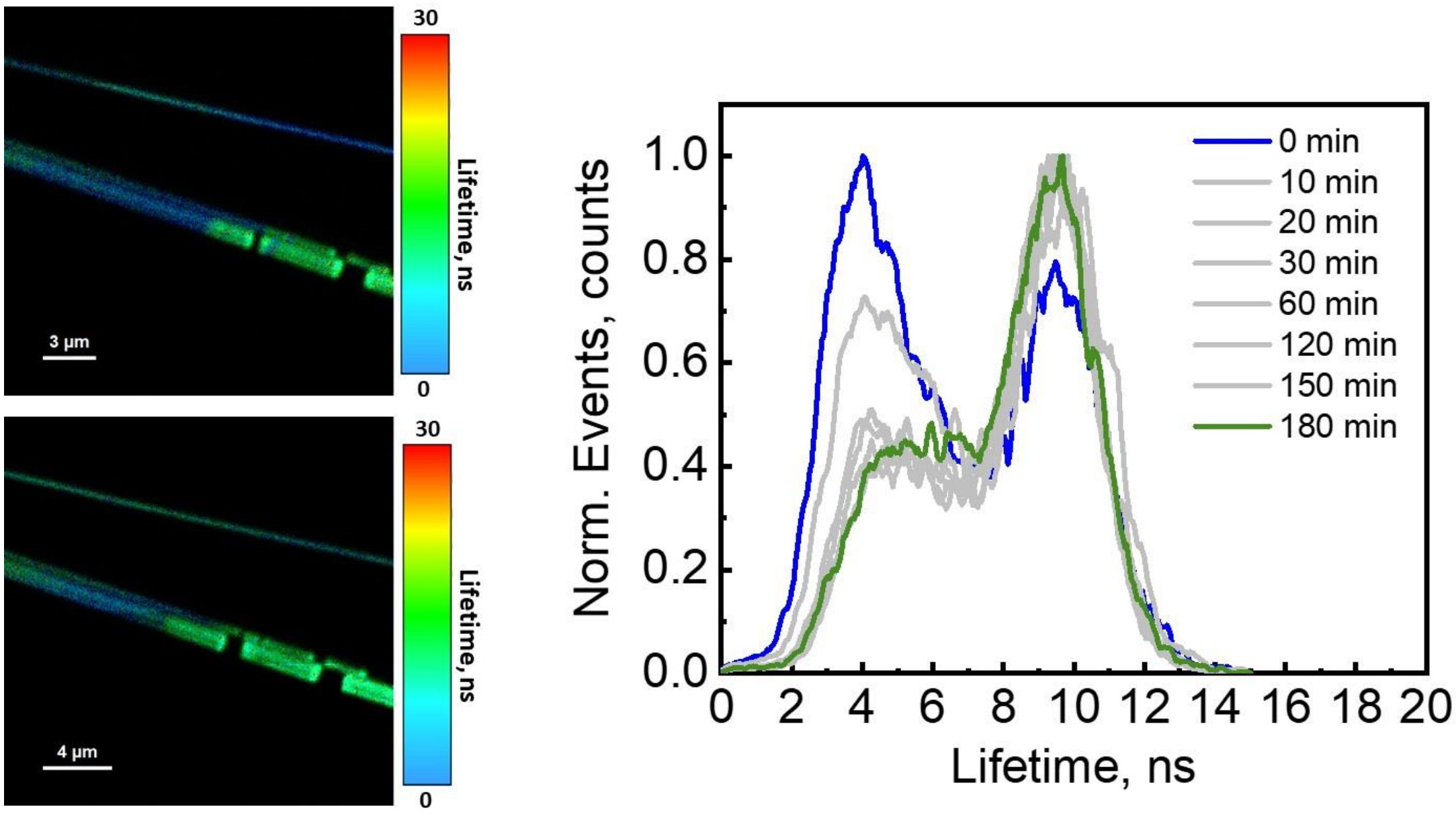

Figure S1: Time-dependent FLIM measurements of the Cy-PDI_2 aggregate fibrils, monitored from 0 to 180 minutes. The FLIM images clearly depict the phase propagation in the aggregate fibrils and the fluorescence lifetime distribution clearly indicates the transformation from blue phase to green phase.

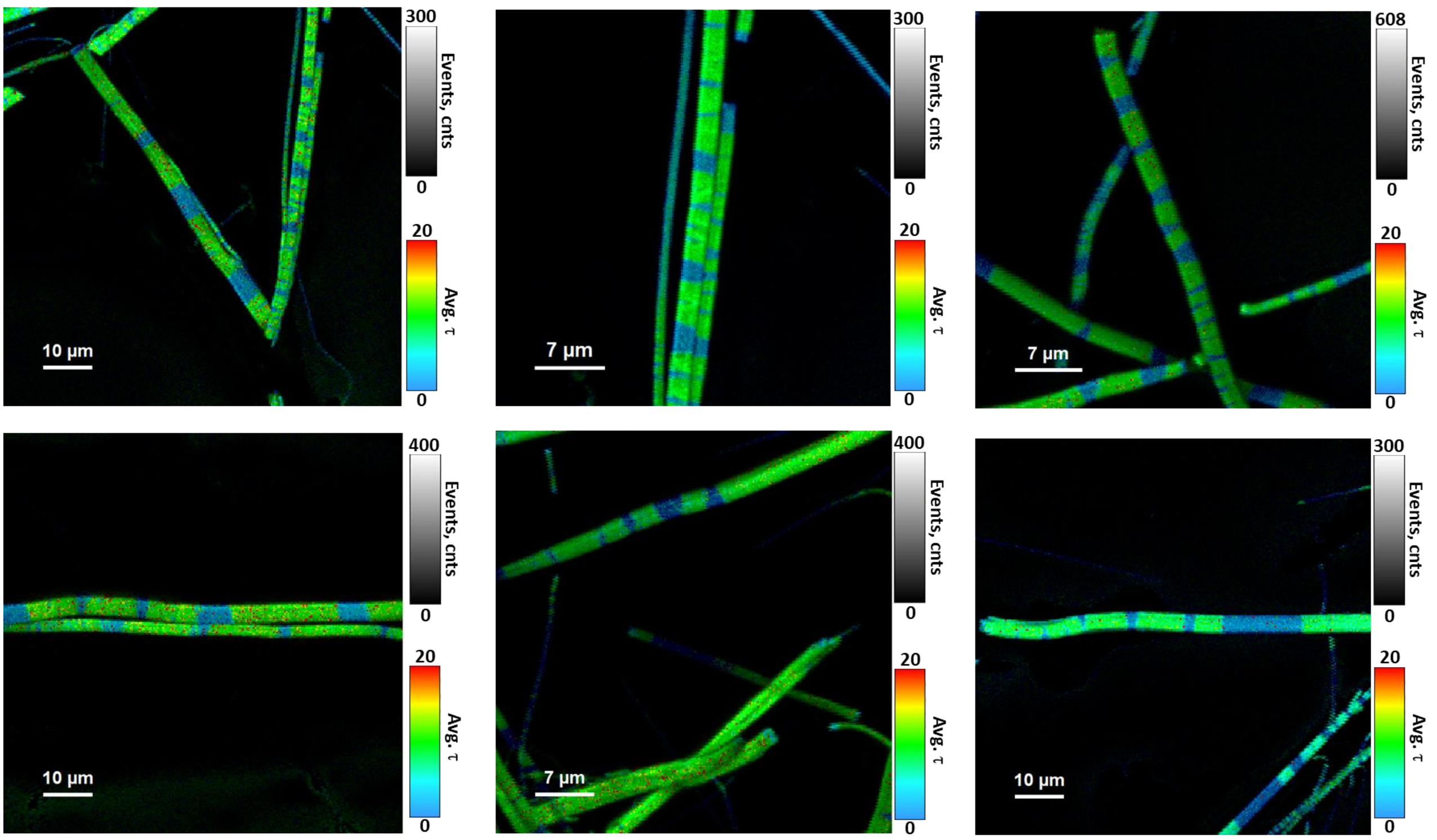

Figure S2: Representative FLIM images of the aggregates of RCy-PDI_2 in toluene drop-casted on a coverslip surface.

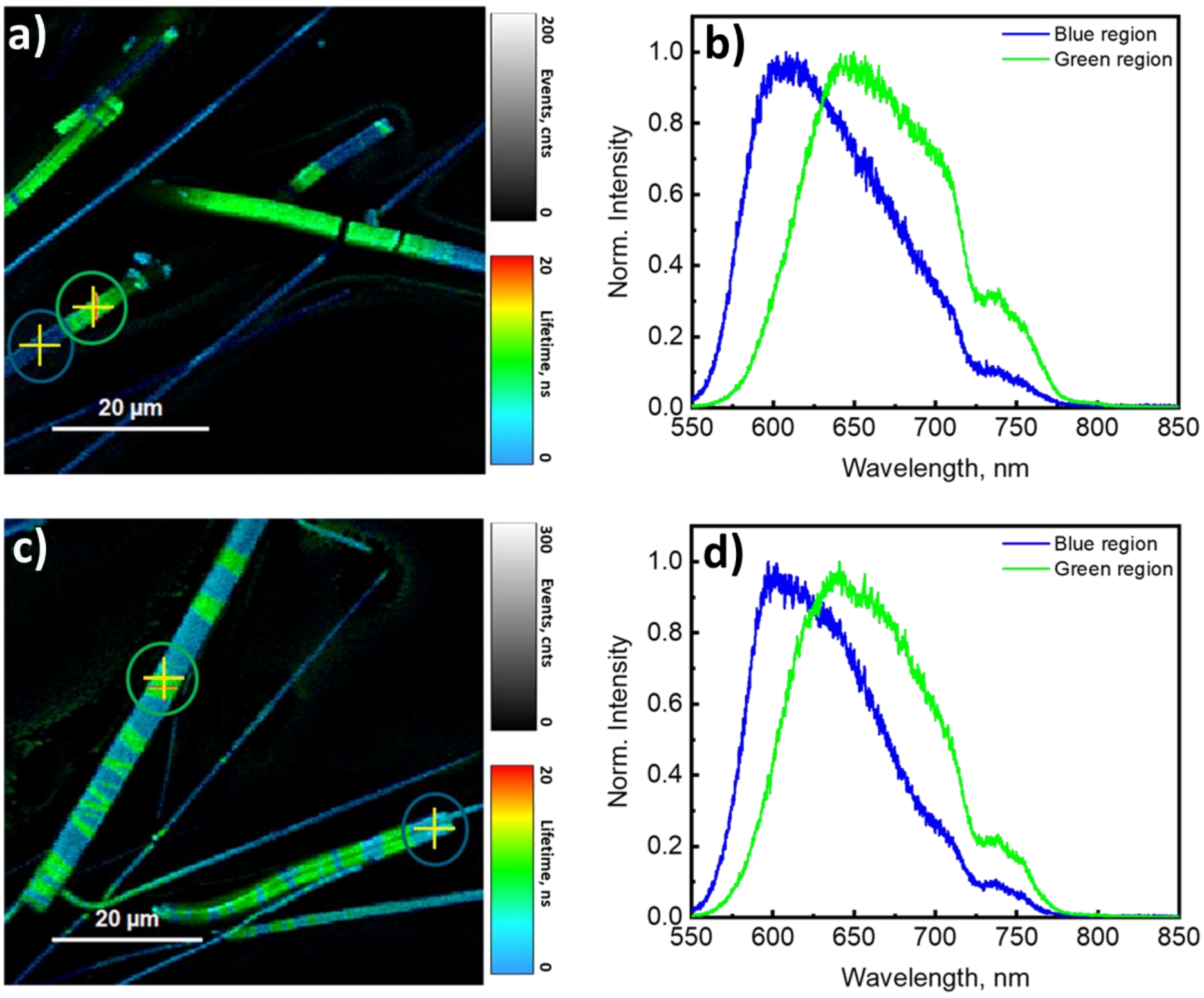

Figure S3: Representative FLIM images of ribbon-like aggregates of RCy-PDI_2 formed in TOL (a, c) and the corresponding emission spectra recorded from the spectrograph (b, d). The blue and green circles with plus show the regions of interest within the aggregates from which the respective emission spectrum was obtained.

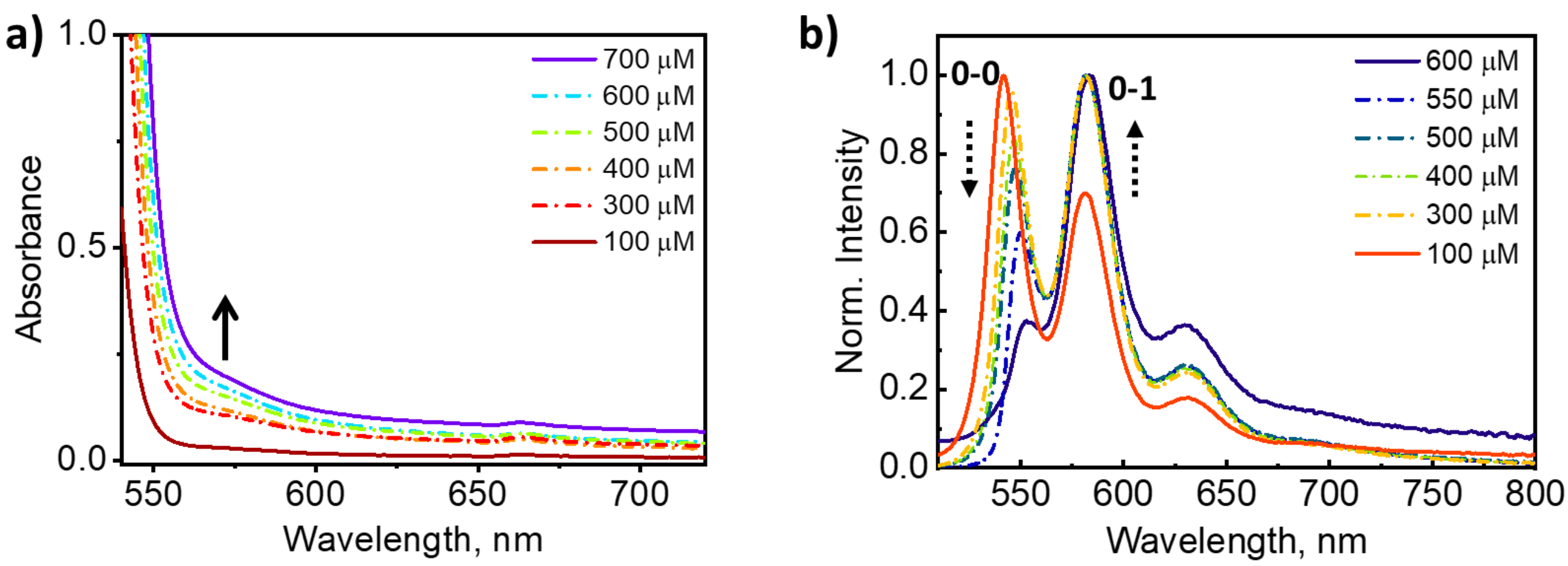

Figure S4: a) Concentration-dependent steady-state absorption and b) fluorescence emission measurements of RCy-PDI_2 in toluene.

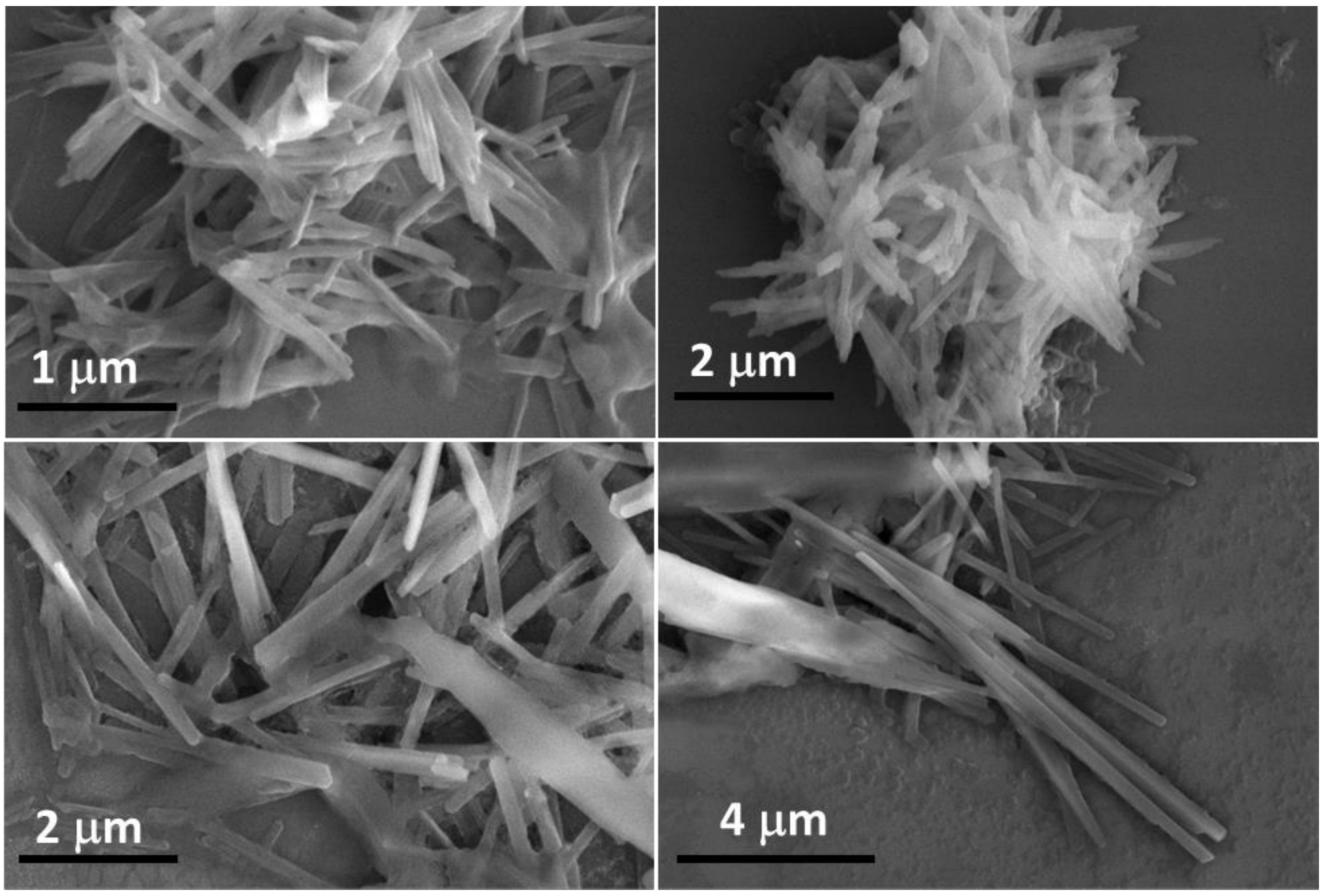

Figure S5: SEM images of the RCy-PDI_2 aggregates in TOL showing diverse fibrils, ribbon-like structures, and some complex secondary structures formed from fibrils.

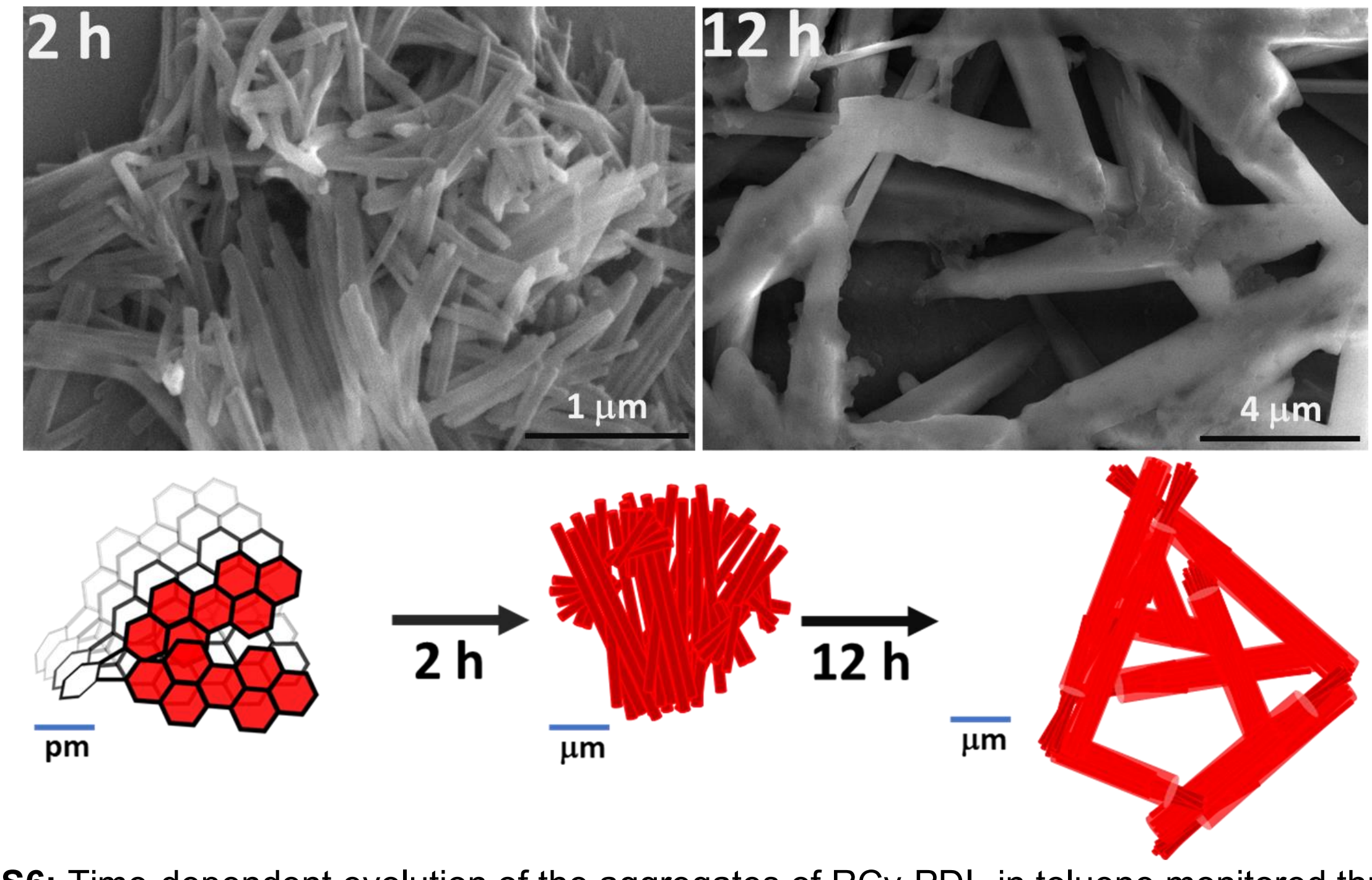

Figure S6: Time-dependent evolution of the aggregates of RCy-PDI_2 in toluene monitored through the SEM imaging technique.

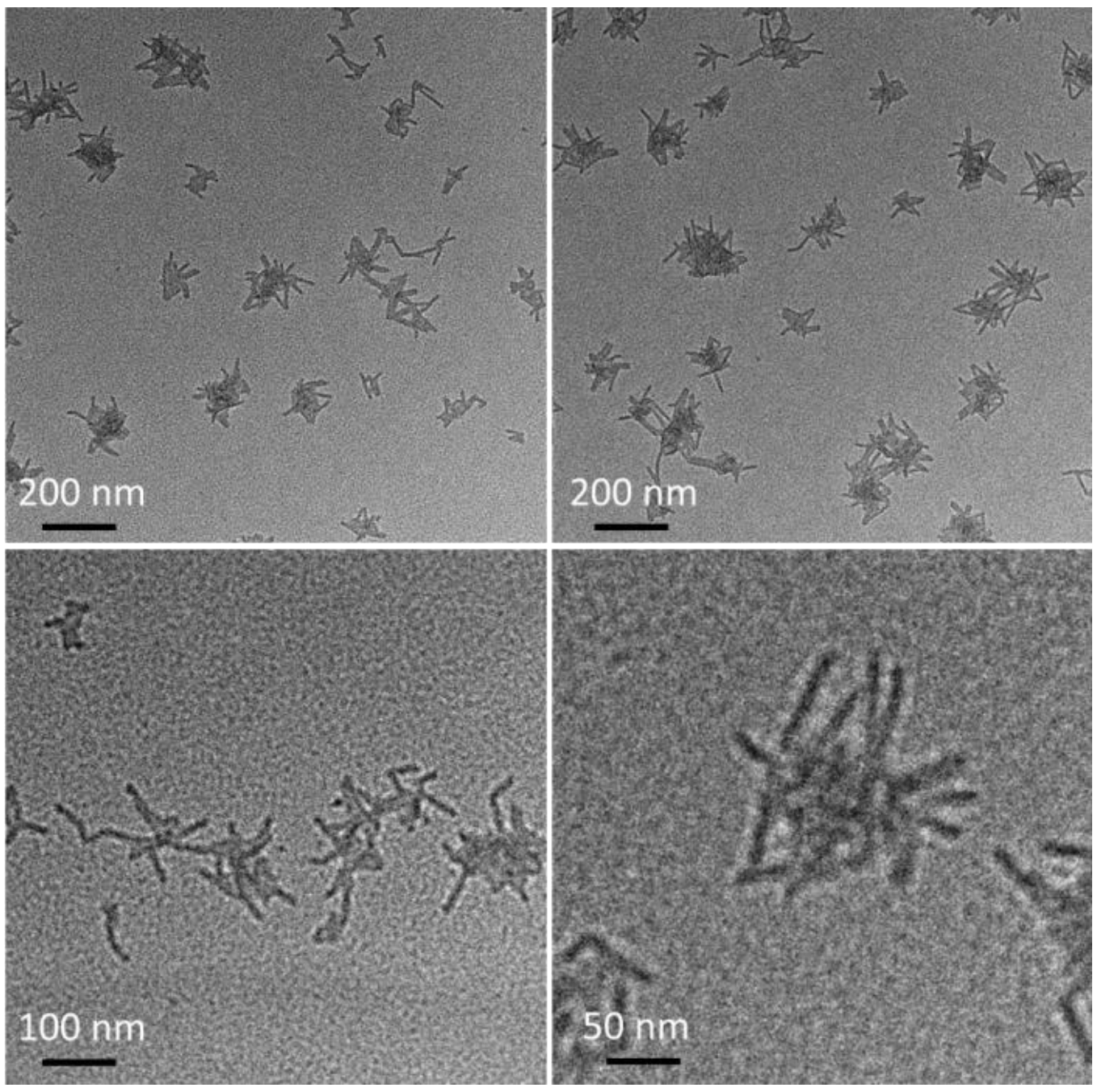

Figure S7: TEM images of the aggregates show that small fibril-like structures are formed, which seed the growth of bigger ribbon-like structures.

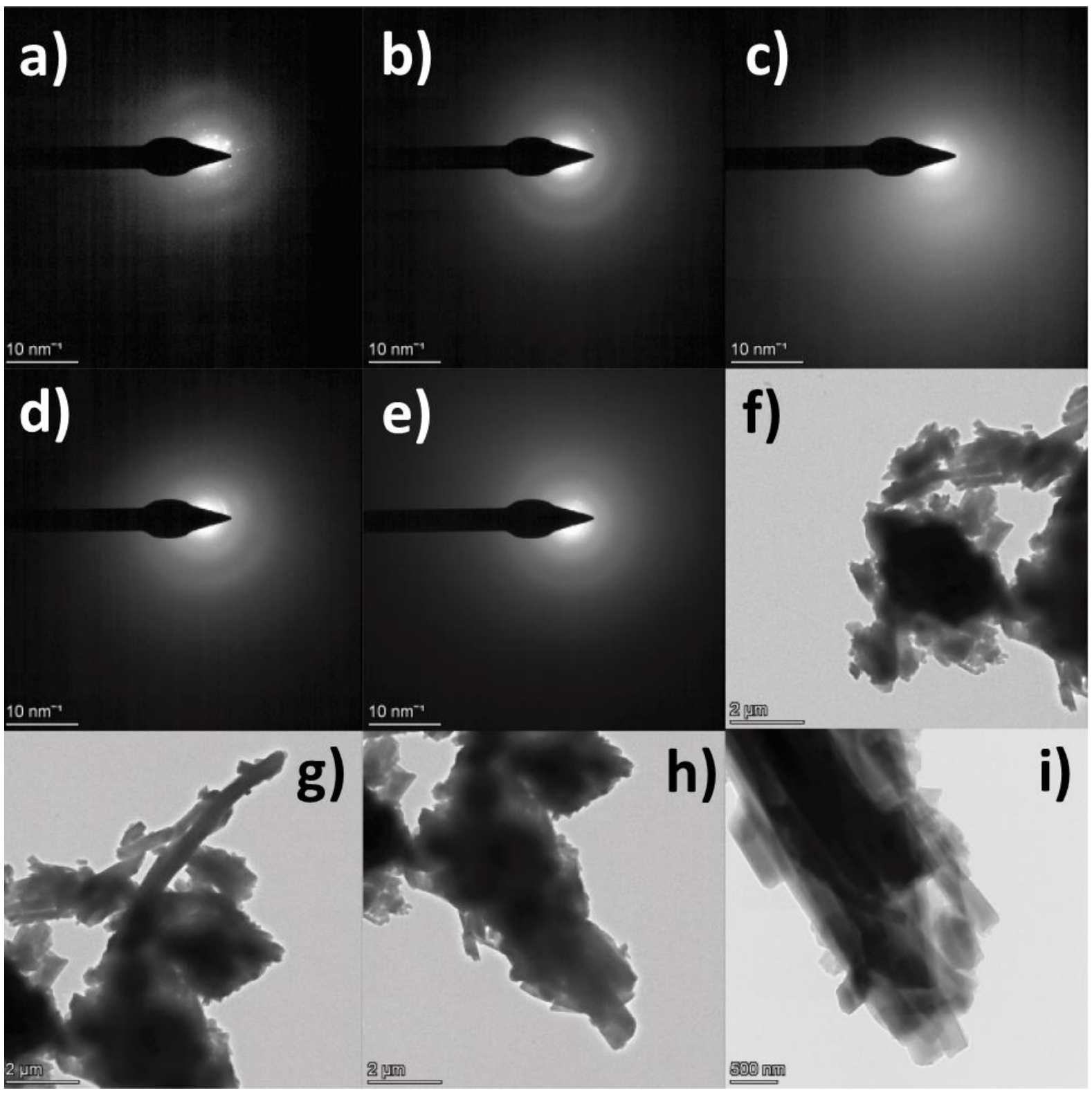

Figure S8: a)- e) The TEM diffraction pattern and f)-i) TEM images of the aggregate superstructures along with the scale. The TEM measurements were carried out to determine the crystallinity for the 3D ED measurements.

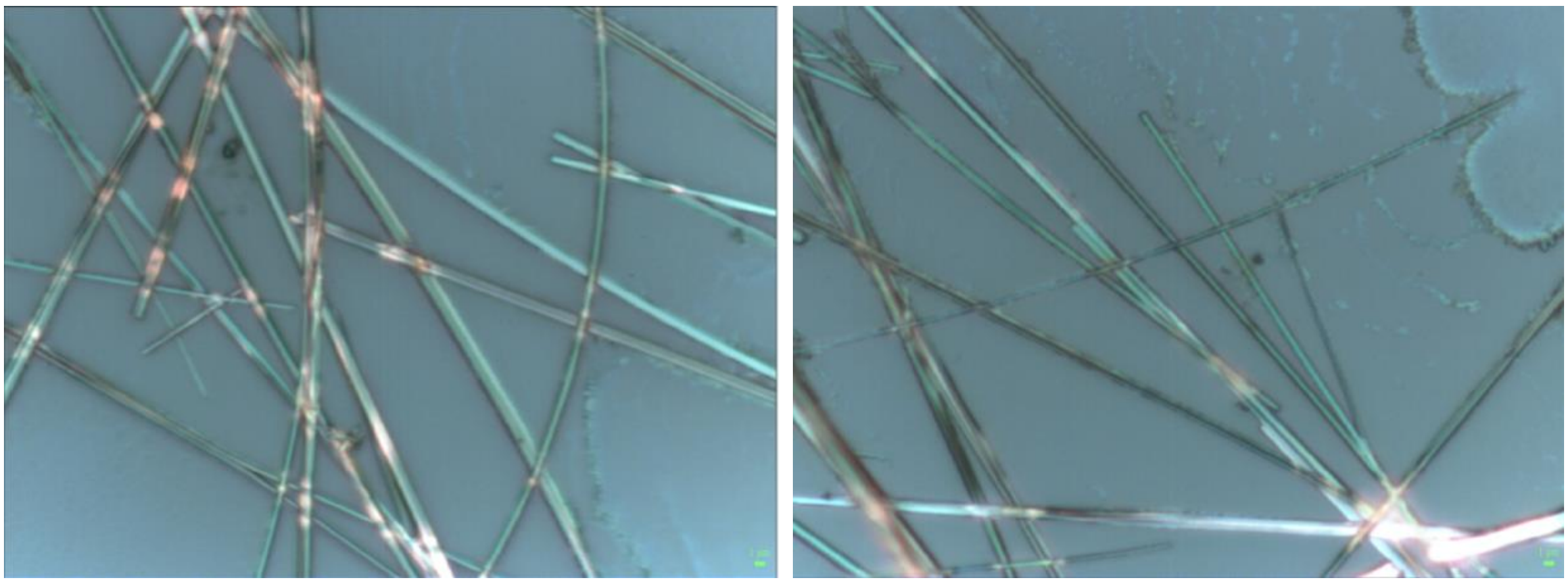

Figure S9: The confocal microscopy images of RCy-PDI$_2$ aggregate fibres with 100x magnification showing distinct bands upon white light excitation.

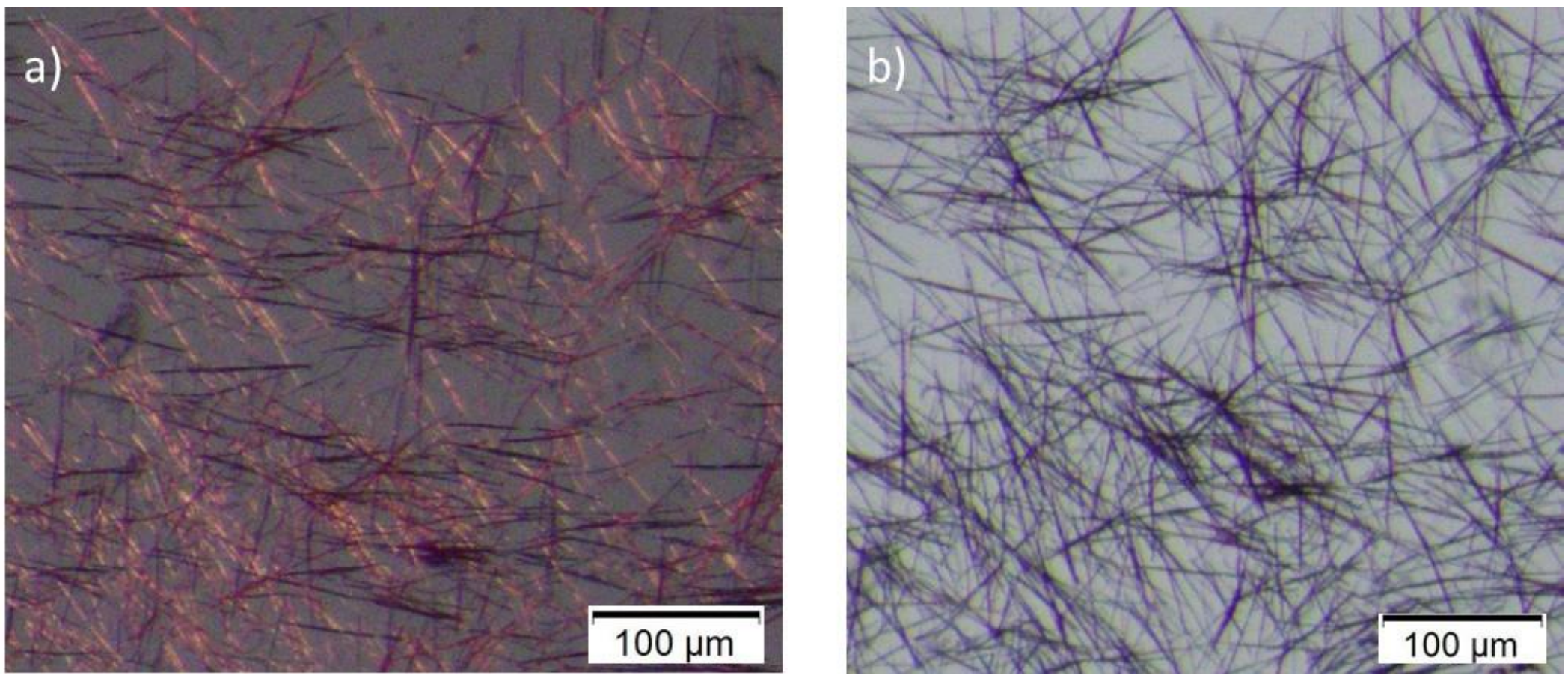

Figure S10: Polarization microscopy images of the RCy-PDI$_2$ aggregate fibrils with a) perpendicular and b) parallel orientation of the polarizer.

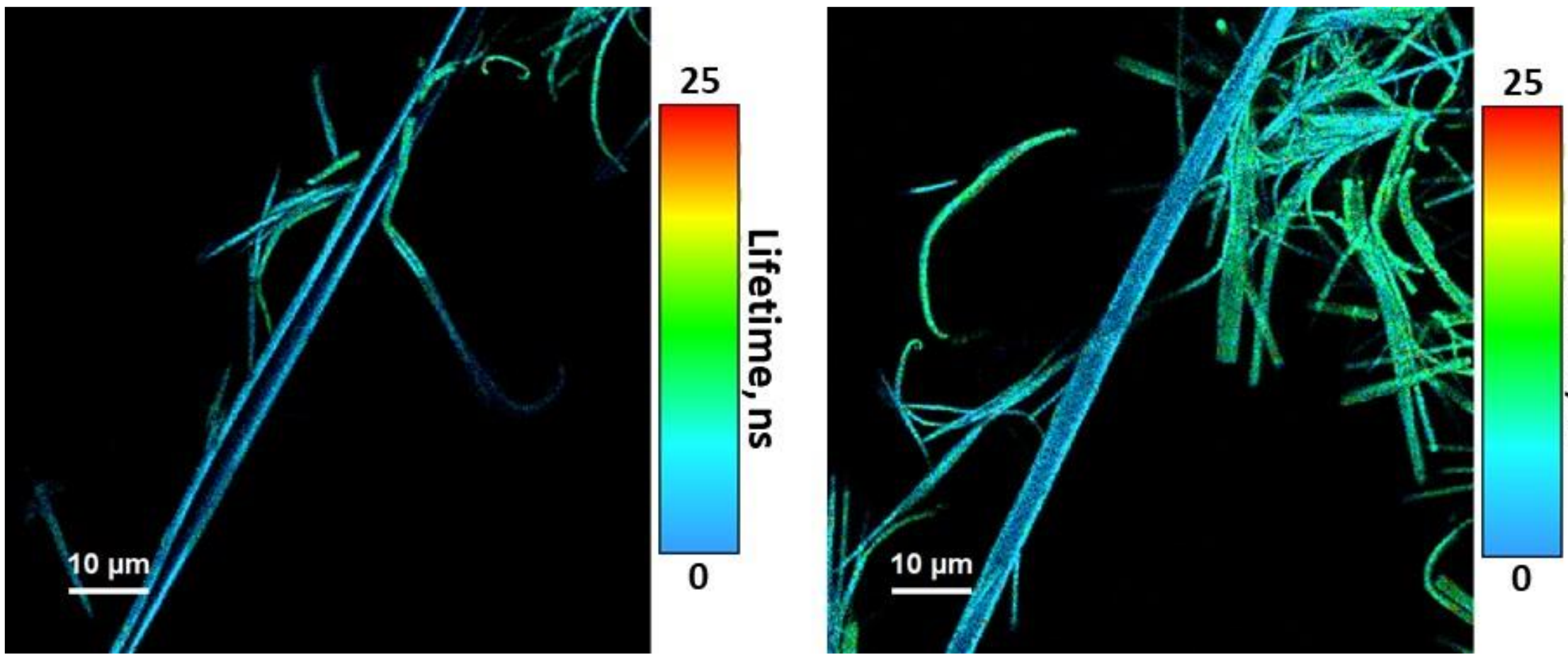

Figure S11: The supernatant solution was boiled at 140 °C until all the particles of the molecules were completely dissolved. The RCy-PDI$_2$ solution was systematically cooled with 10 °C interval until 60 °C. Small aggregate fibrils observable to naked eyes starts to develop in the aggregate solution. The RCy-PDI$_2$ aggregate solution is cooled to room temperature and drop-casted freshly for the FLIM measurements.

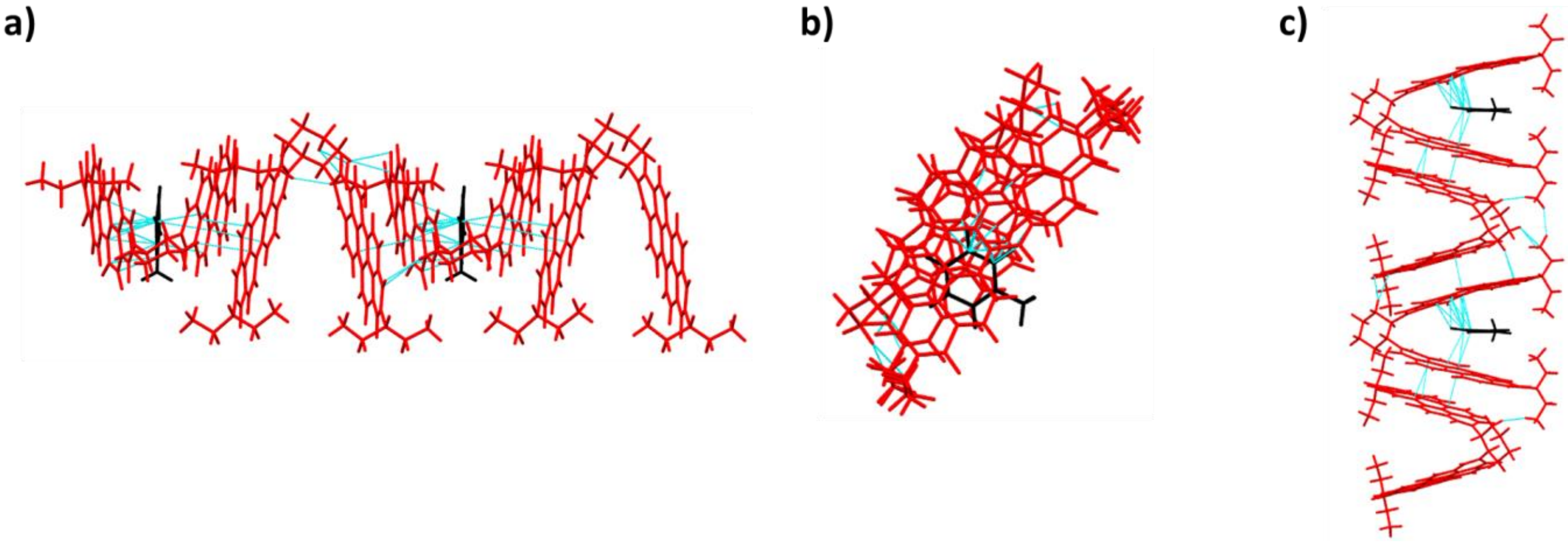

Figure S12: The crystal structure for the RCy-PDI$_2$ crystalline aggregate obtained from 3D ED shown along a) a-axis, b) b-axis and c) c-axis.

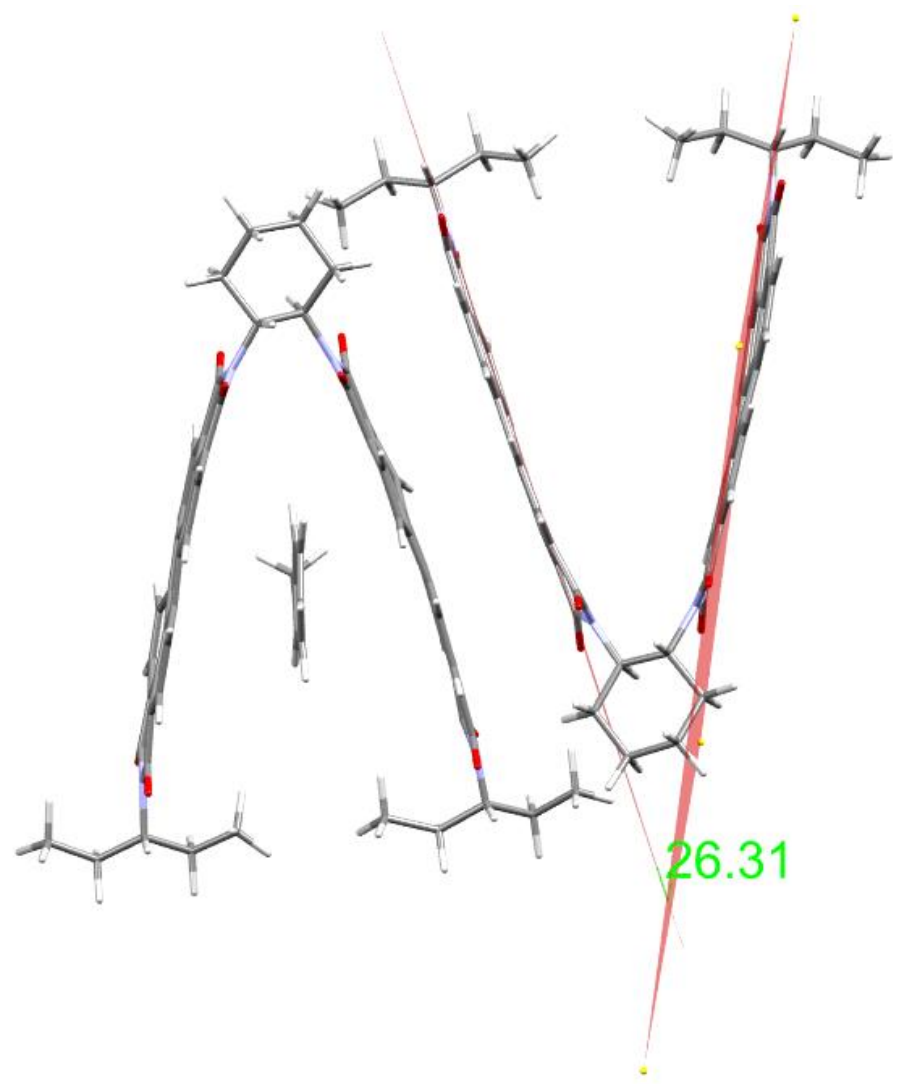

Figure S13: The interplanar angle between the two PDI units of the RCy-PDI$_2$ solved structure. The angle is measured at the intersection of the two planes.

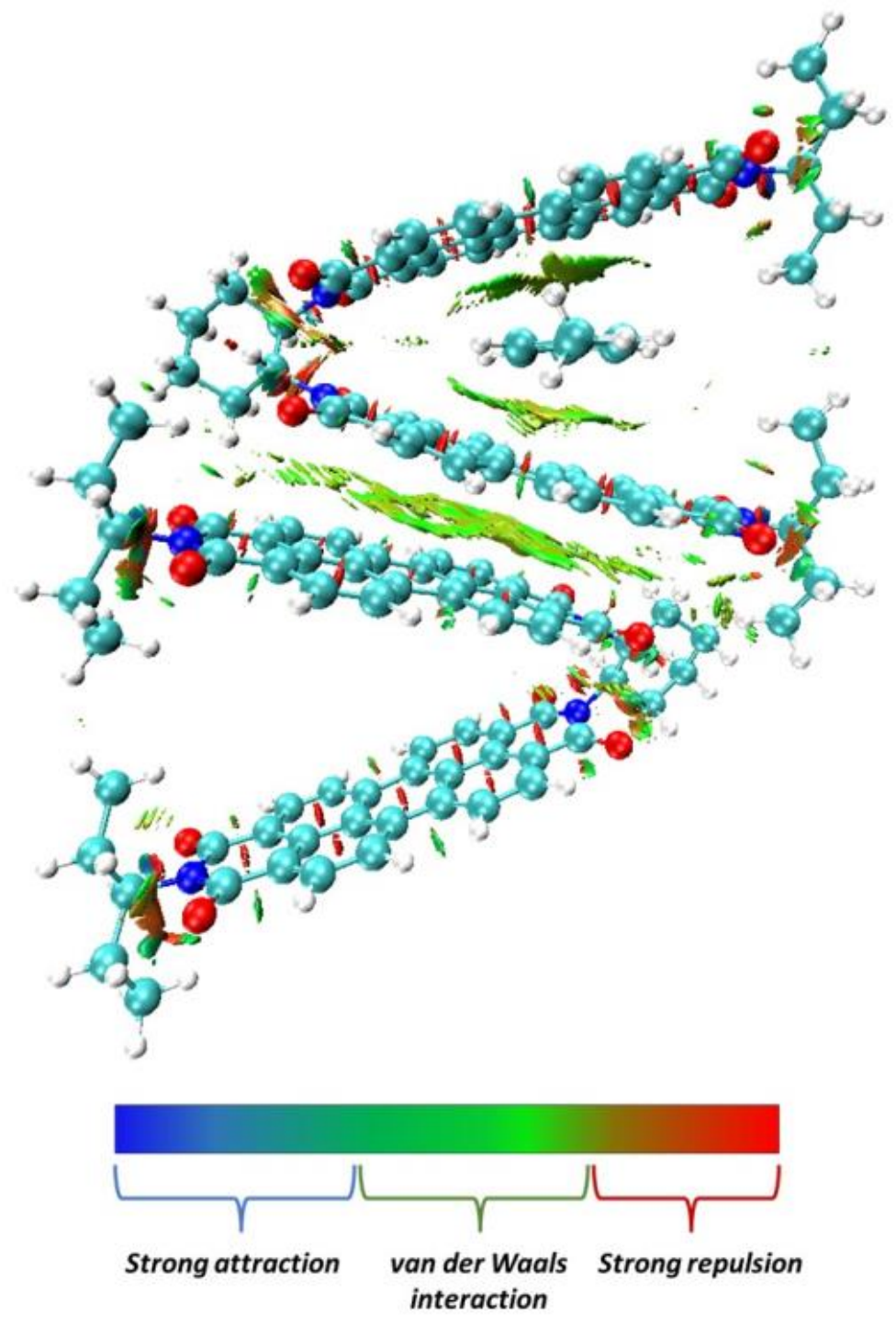

Figure S14: NCI analysis of the RCy-PDI$_2$ aggregate structure obtained from the 3D ED measurement. The prominent π-π interaction between the PDI units intermolecularly and the toluene-stacked PDI units also has an enhanced π-π interaction attributed to the J-type coupling in the aggregate.

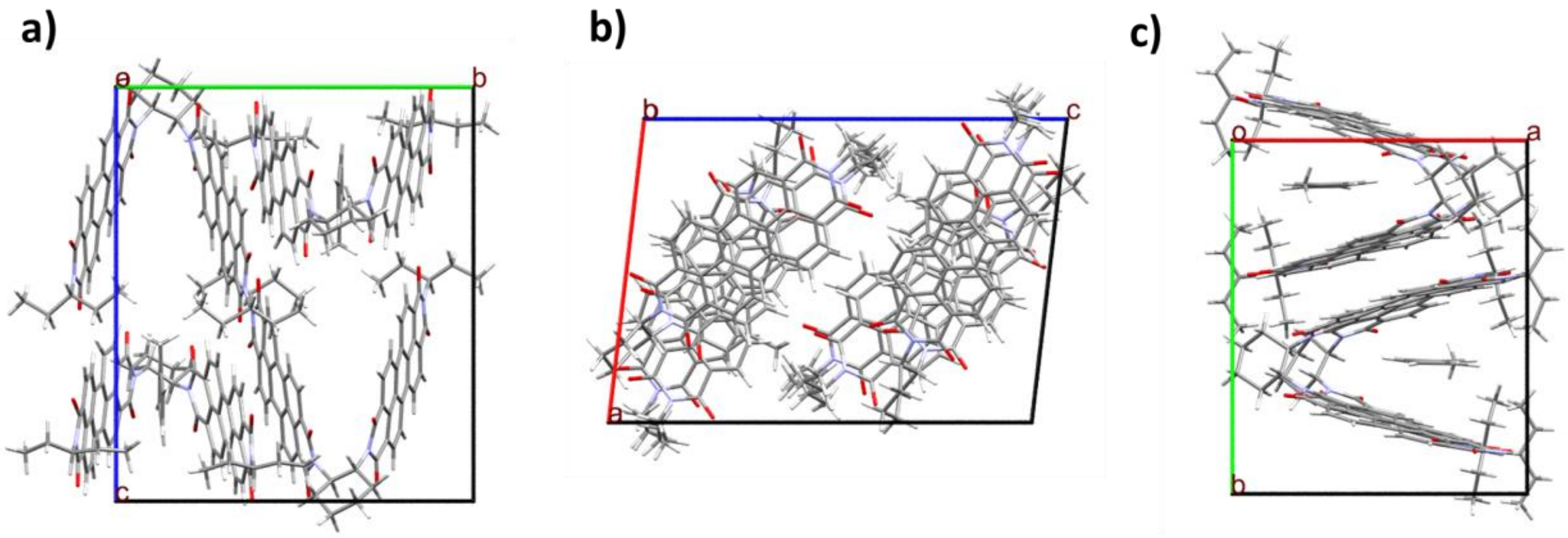

Figure S15: The representative packing of the aggregate in a) along a a-axis, which gives the perspective of alternating orientation of the RCy-PDI_2 molecules, b) along the b-axis, which gives the view of π-π stacking in the RCy-PDI_2 molecules, and c) along the c-axis of the crystalline fibre which shows the prominent interaction of the intermolecular PDI units responsible for the excimer formation.

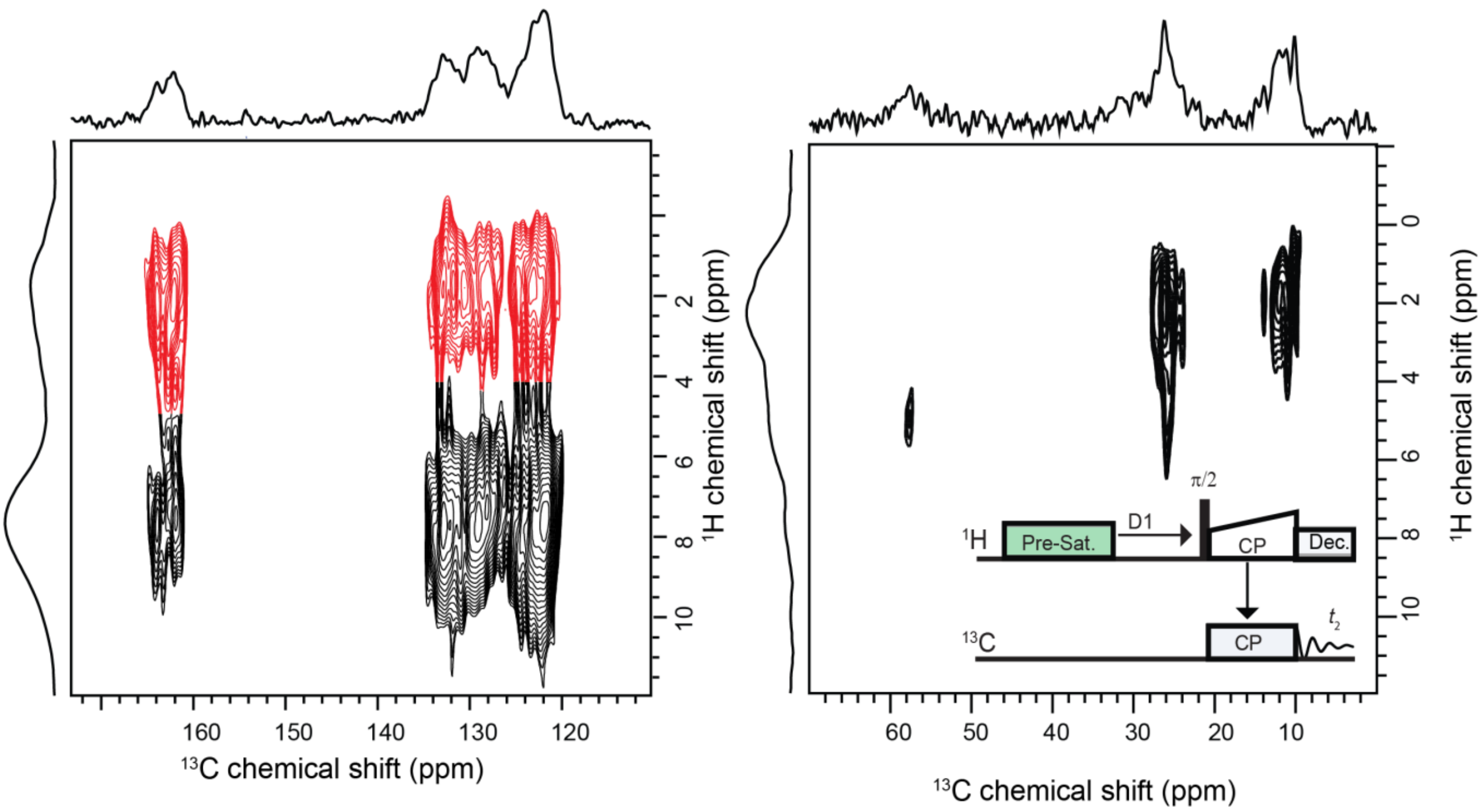

Figure S16: ^1H-^{13}C HETCOR spectra of the RCy-PDI_2 aggregate in solid state collected at long contact time**.**

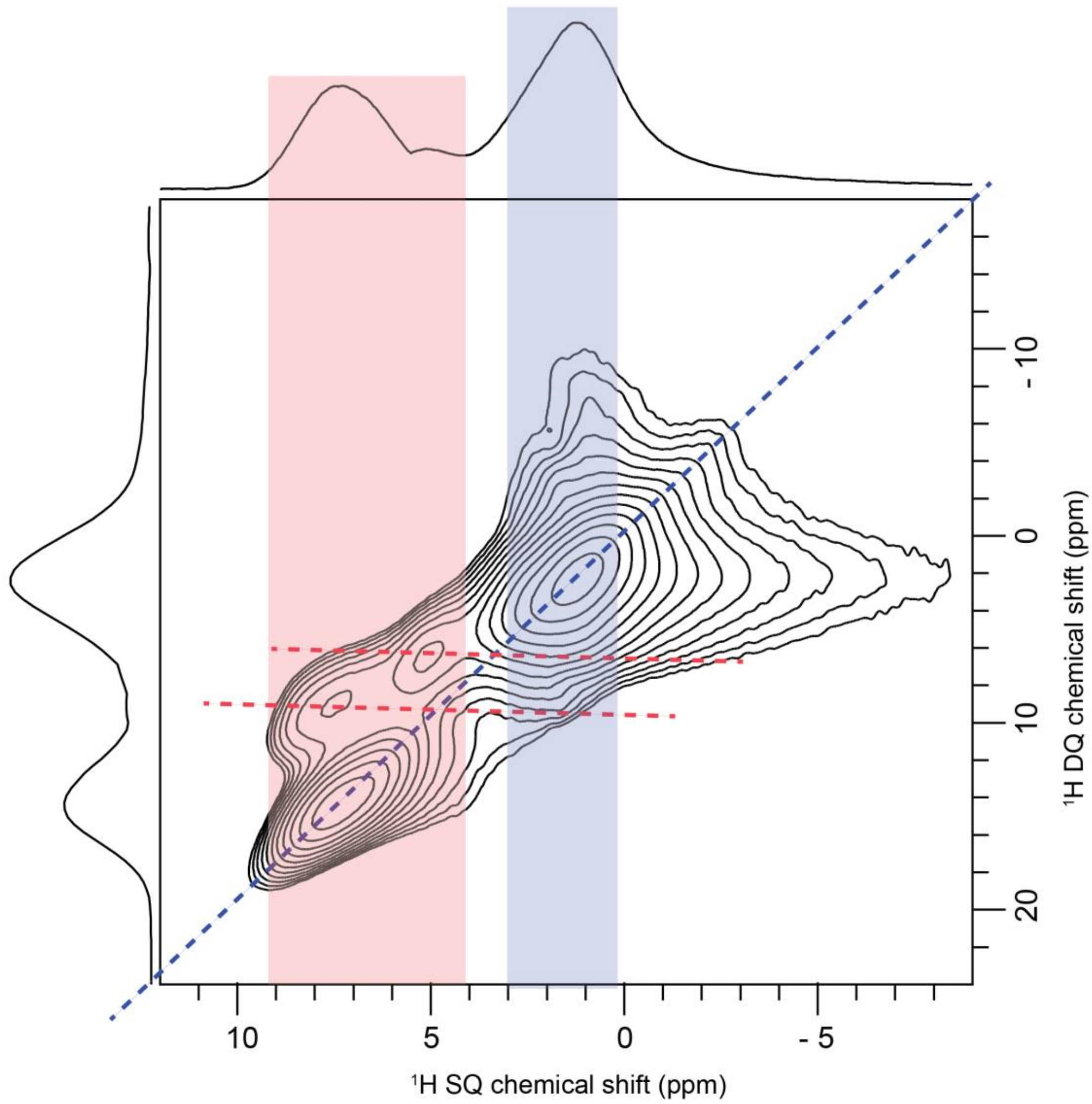

Figure S17: The ^1H-^1H BABA spectrum of the RCy-PDI_2 aggregate in solid state collected at 60 kHz spinning speed. The arrows represent the correlation peaks between the aliphatic and aromatic peaks in the 2D spectrum.

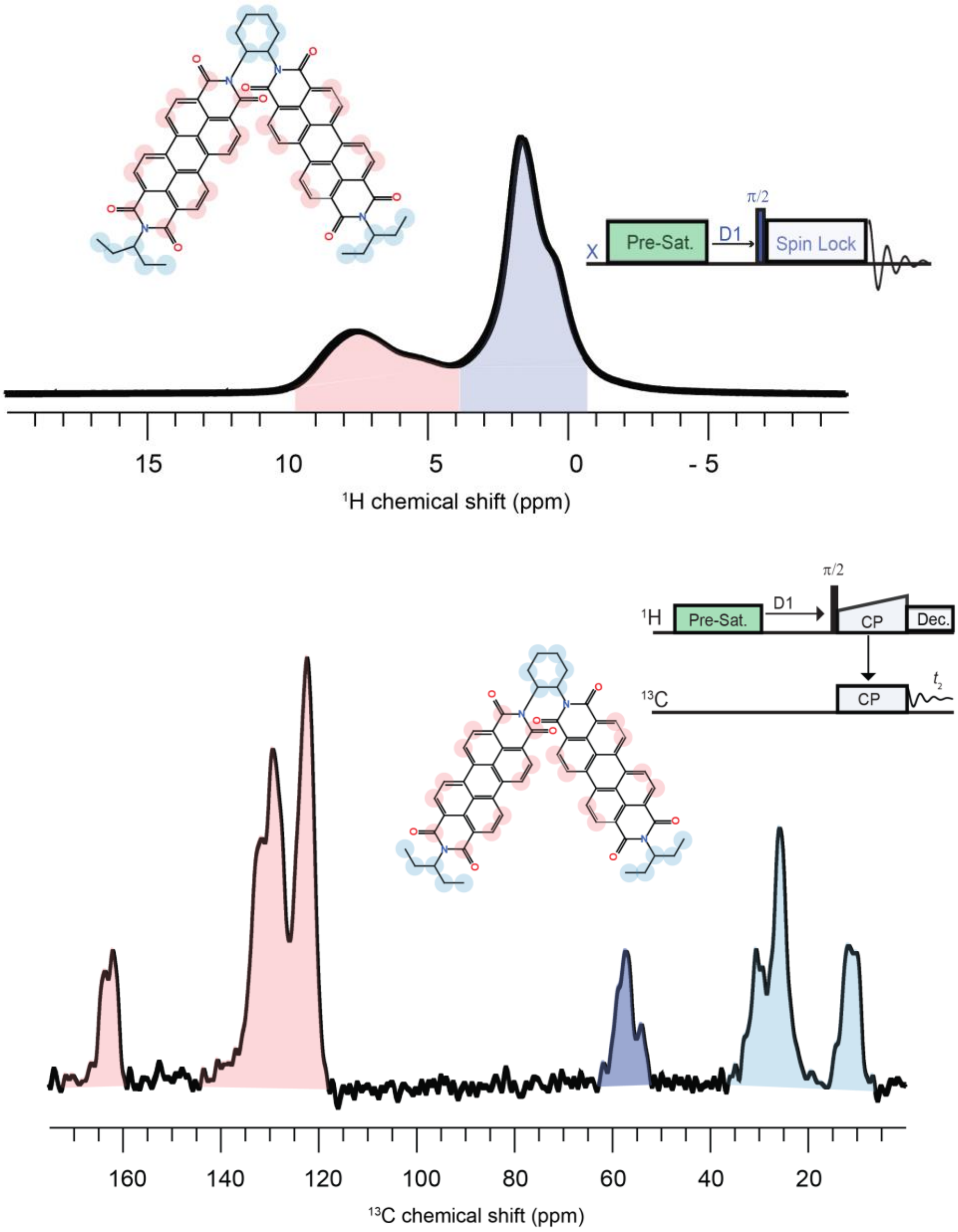

Figure S18: The ^{1}H NMR spectra of the $RCyPDI_2$ collected at 60 kHz, along with the 1D ^{13}C CP solid state NMR spectra.

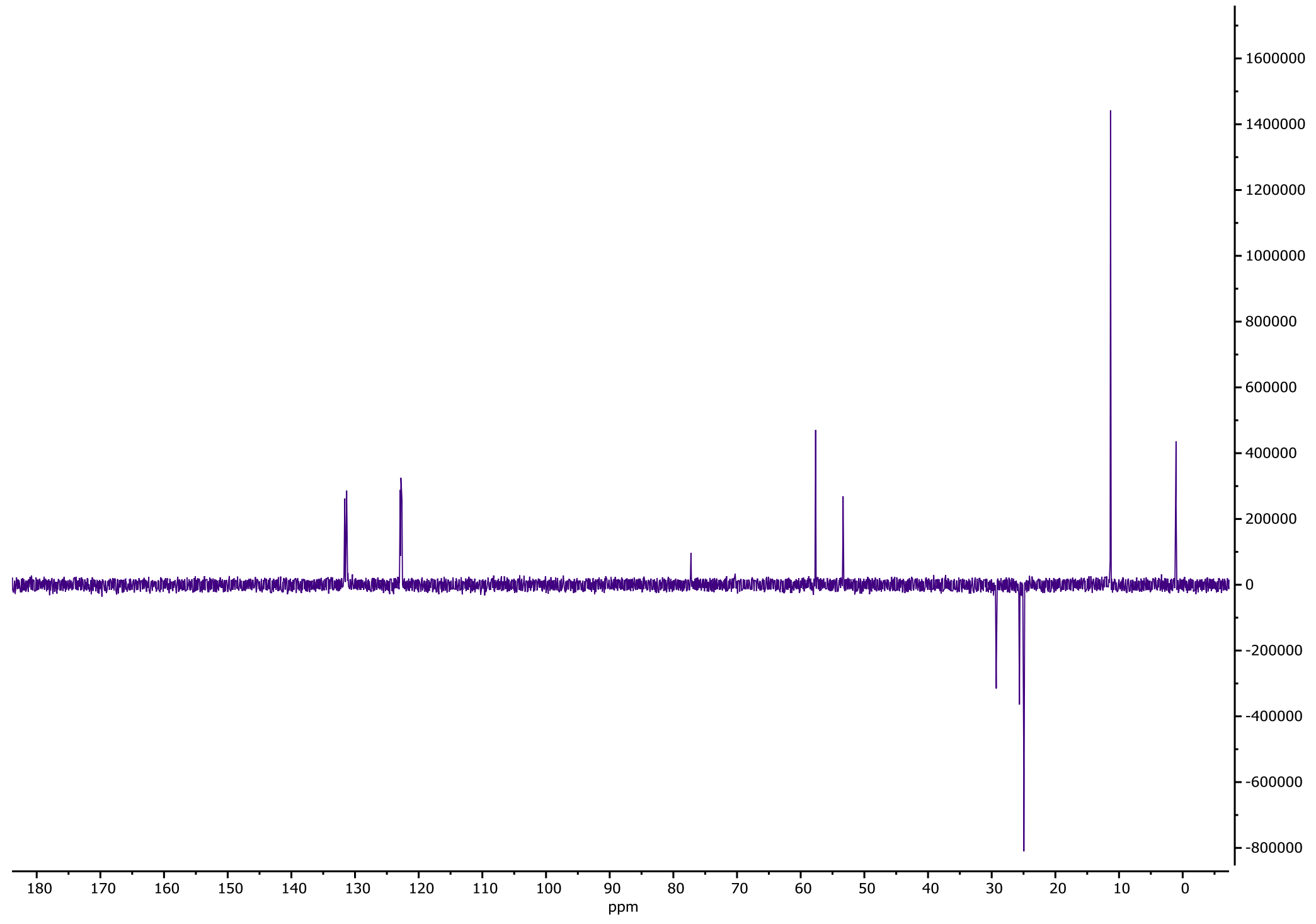

Figure S19: ^{13}C DEPT 135 spectrum of the RCy-PDI_2 molecule in $CDCl_3$.

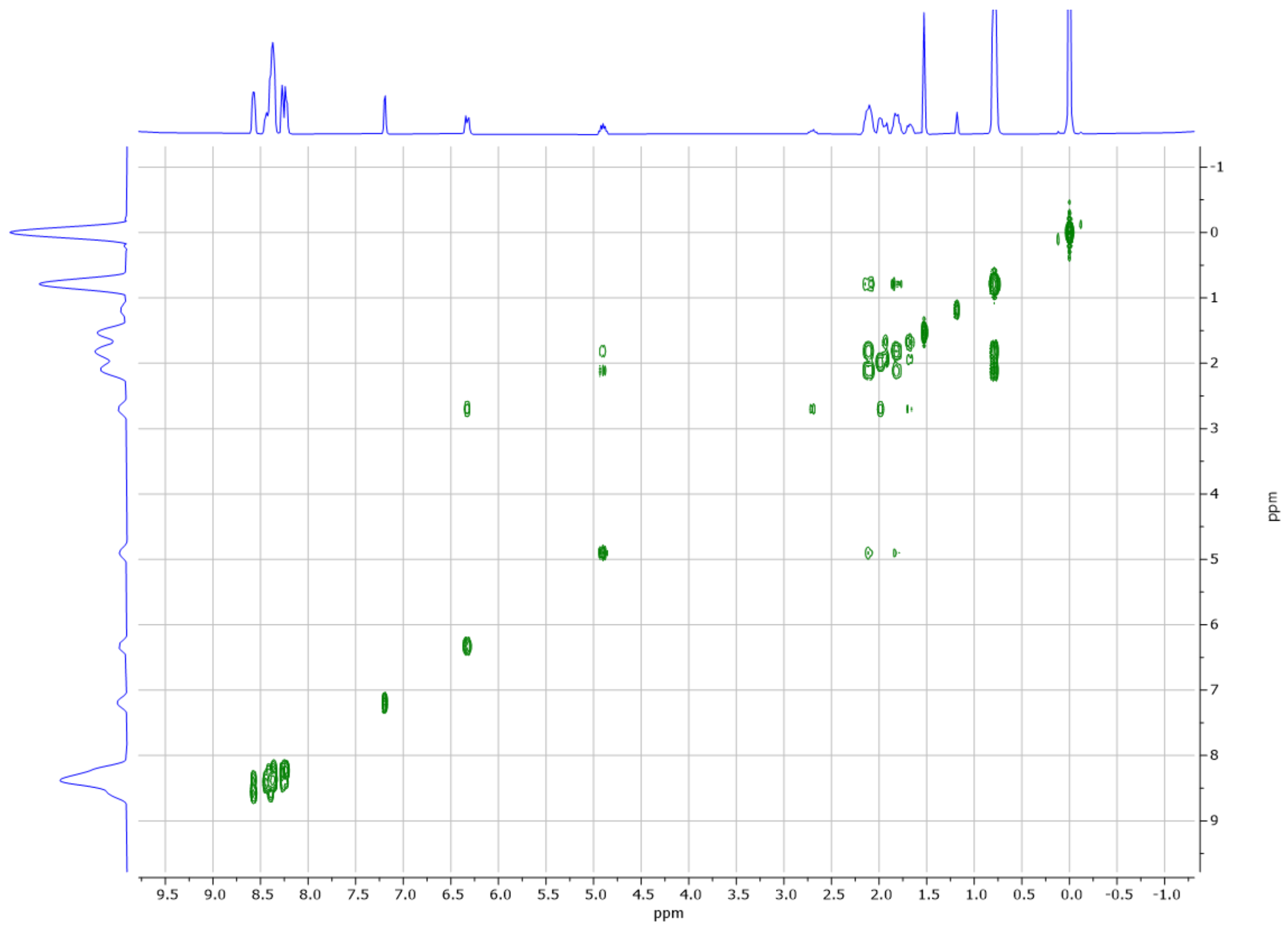

Figure S20: The ^{1}H-^{1}H COSY spectrum of the RCy-PDI_2 molecule in $CDCl_3$.

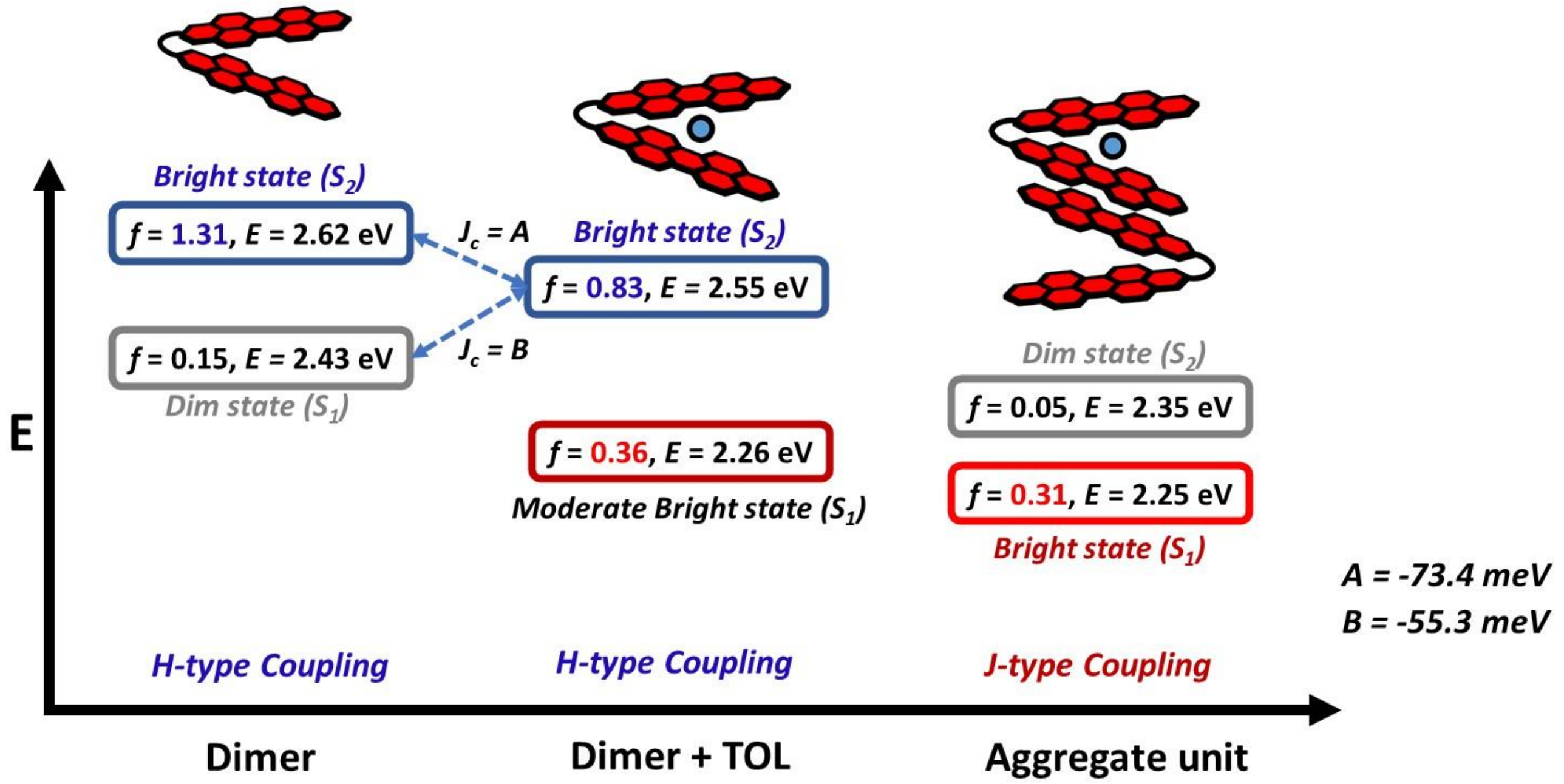

Figure S21: The schematic representation of the energy profiles calculated using TDDFT calculations in Gaussian 16.1 for the RCy-PDI_2 molecule, where the Dimer structure coordinates are considered from the crystal structure and the Dimer intercalated with a toluene molecule is also evaluated and compared with the unit cell structure (Dimer and Dimer with toluene). The lower energy bright state of the aggregate unit confirms the J-type coupling in RCy-PDI_2 aggregate. EET calculations show strong J-type coupling between the two bright states and energetically proximal states of the dimer fragments considered for the calculation.

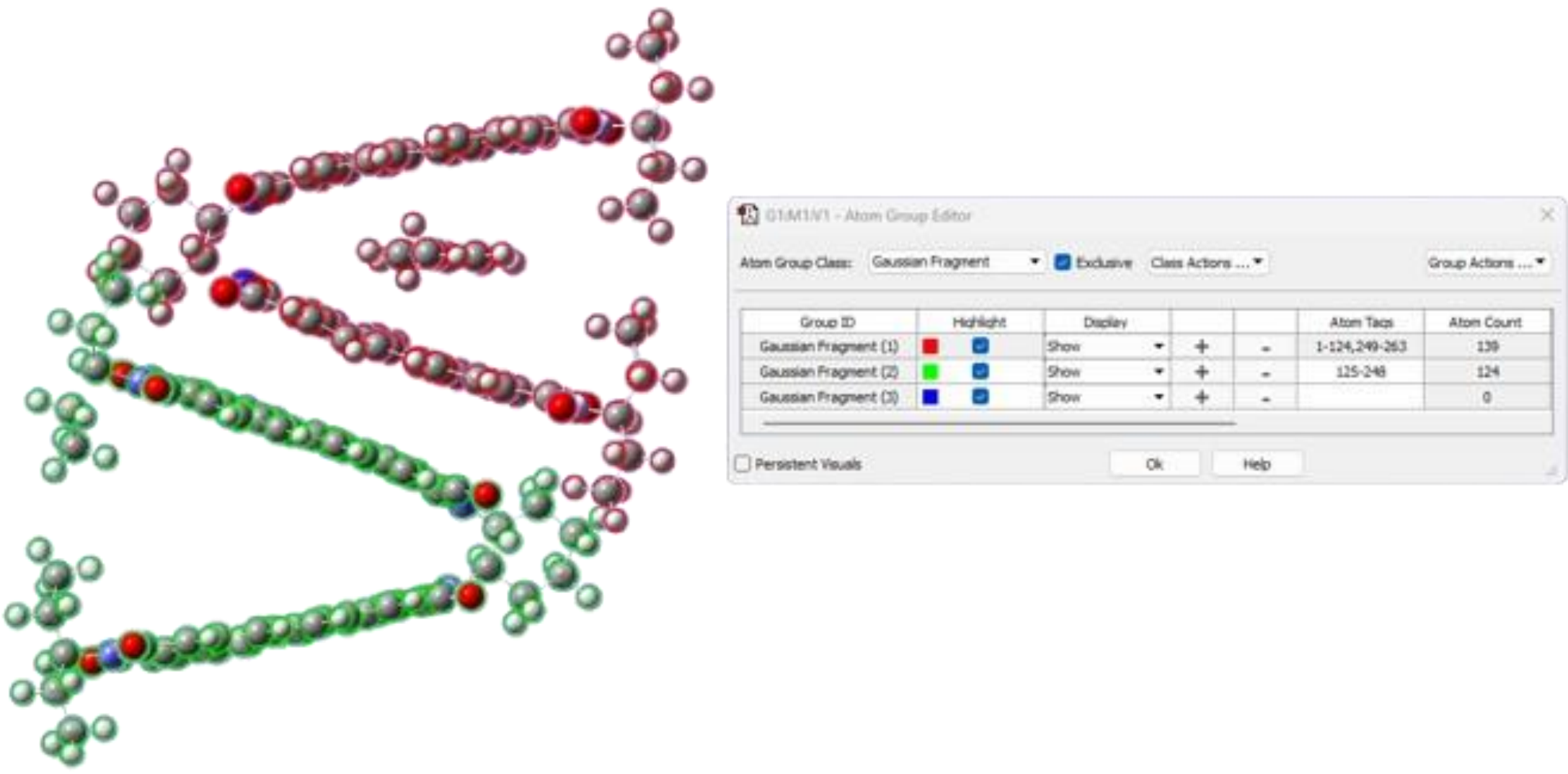

Figure S22: The Gaussian fragments employed for the EET calculations of the aggregate. The toluene intercalated structure is fragment 1(red highlight) and the toluene detrapped dimer is assigned as fragment 2 (green highlight).

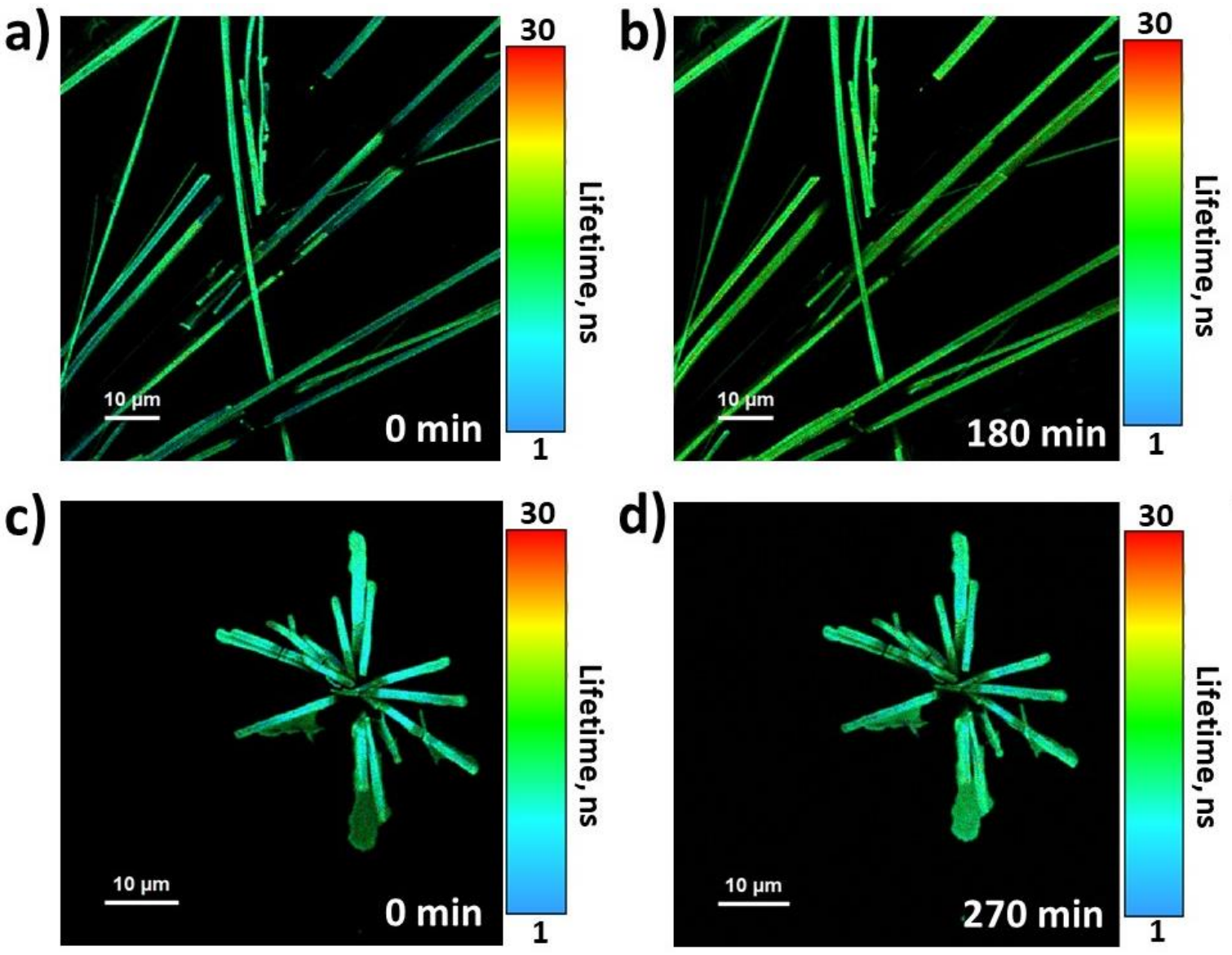

e)

In Benzene

Time (min)	Population (B%/G%)	Blue (ns)	Green (ns)
0	38/62	2.9	10.8
180	19/81	2.3	13

f)

In o-Xylene

Time (min)	Population (B%/G%)	Blue (ns)	Green (ns)
0	48/51	1.9	10.4
270	47/52	1.7	11

Figure S23: The time-dependent FLIM measurements of RCy-PDI_2 aggregates in benzene from a) 0 min to b) 180 min. Time-dependent FLIM measurements of the RCy-PDI_2 aggregates in o-xylene at c) 0 min and b) 270 min. The fluorescence lifetime population of the long and short fluorescence lifetime components of the FLIM acquired at different time frames of the aggregates formed in e) benzene and f) o-xylene.

Reference

(1) Maret, P. D.; Sasikumar, D.; Sebastian, E.; Hariharan, M. Symmetry-Breaking Charge Separation in a Chiral Bis(Perylenediimide) Probed at Ensemble and Single-Molecule Levels. *J. Phys. Chem. Lett.* **2023**, *14* (38), 8667–8675. https://doi.org/10.1021/acs.jpclett.3c01889.

(2) Gaussian 16, Revision C.01, M. J. Frisch, G. W. Trucks, H. B. Schlegel, G. E. Scuseria, M. A. Robb, J. R. Cheeseman, G. Scalmani, V. Barone, G. A. Petersson, H. Nakatsuji, X. Li, M. Caricato, A. V. Marenich, J. Bloino, B. G. Janesko, R. Gomperts, B. Mennucci, H. P. Hratchian, J. V. Ortiz, A. F. Izmaylov, J. L. Sonnenberg, D. Williams-Young, F. Ding, F. Lipparini, F. Egidi, J. Goings, B. Peng, A. Petrone, T. Henderson, D. Ranasinghe, V. G. Zakrzewski, J. Gao, N. Rega, G. Zheng, W. Liang, M. Hada, M. Ehara, K. Toyota, R. Fukuda, J. Hasegawa, M. Ishida, T. Nakajima, Y. Honda, O. Kitao, H. Nakai, T. Vreven, K. Throssell, J. A. Montgomery, Jr., J. E. Peralta, F. Ogliaro, M. J. Bearpark, J. J. Heyd, E. N. Brothers, K. N. Kudin, V. N. Staroverov, T. A. Keith, R. Kobayashi, J. Normand, K. Raghavachari, A. P. Rendell, J. C. Burant, S. S. Iyengar, J. Tomasi, M. Cossi, J. M. Millam, M. Klene, C. Adamo, R. Cammi, J. W. Ochterski, R. L. Martin, K. Morokuma, O. Farkas, J. B. Foresman, and D. J. Fox, Gaussian, Inc., Wallingford CT, 2016.

(3) ELDIX Software Suite, Version 5.24.0; ELDICO Scientific AG: Allschwil, Switzerland, 2025.

(4) Apex Suite of Crystallographic Software, APEX4, Version 2022.1-1; Bruker AXS Inc.: Madison, Wisconsin, USA, 2022.

(5) SAINT, Version 8.40B; Bruker AXS Inc.: Madison, Wisconsin, USA,2019.

(6) SADABS, Version 2016/2; Bruker AXS Inc.: Madison, Wisconsin, USA, 2016.

(7) Sheldrick, G. M. Experimental Phasing with *SHELXC* / *D* / *E* : Combining Chain Tracing with Density Modification. *Acta Crystallogr D Biol Crystallogr* **2010**, *66* (4), 479–485. https://doi.org/10.1107/S0907444909038360.

(8) Sheldrick, G. M. Crystal Structure Refinement with *SHELXL*. *Acta Crystallogr C Struct Chem* **2015**, *71* (1), 3–8. https://doi.org/10.1107/S2053229614024218.

(9) Hübschle, C. B.; Sheldrick, G. M.; Dittrich, B. *ShelXle* : A Qt Graphical User Interface for *SHELXL*. *J Appl Crystallogr* **2011**, *44* (6), 1281–1284. https://doi.org/10.1107/S0021889811043202.

(10) Dolomanov, O. V.; Bourhis, L. J.; Gildea, R. J.; Howard, J. A. K.; Puschmann, H. *OLEX2* : A Complete Structure Solution, Refinement and Analysis Program. *J Appl Crystallogr* **2009**, *42* (2), 339–341. https://doi.org/10.1107/S0021889808042726.

(11) Peng, L.-M. Electron Atomic Scattering Factors and Scattering Potentials of Crystals. *Micron* **1999**, *30* (6), 625–648. https://doi.org/10.1016/S0968-4328(99)00033-5.

(12) Pickard, C. J.; Mauri, F. All-Electron Magnetic Response with Pseudopotentials: NMR Chemical Shifts. *Phys. Rev. B* **2001**, *63* (24), 245101. https://doi.org/10.1103/PhysRevB.63.245101.

(13) Yates, J. R.; Pickard, C. J.; Mauri, F. Calculation of NMR Chemical Shifts for Extended Systems Using Ultrasoft Pseudopotentials. *Phys. Rev. B* **2007**, *76* (2), 024401. https://doi.org/10.1103/PhysRevB.76.024401.

(14) Perdew, J. P.; Ruzsinszky, A.; Csonka, G. I.; Vydrov, O. A.; Scuseria, G. E.; Constantin, L. A.; Zhou, X.; Burke, K. Restoring the Density-Gradient Expansion for Exchange in Solids and Surfaces. *Phys. Rev. Lett.* **2008**, *100* (13), 136406. https://doi.org/10.1103/PhysRevLett.100.136406.
(15) Tkatchenko, A.; Scheffler, M. Accurate Molecular Van Der Waals Interactions from Ground-State Electron Density and Free-Atom Reference Data. *Phys. Rev. Lett.* **2009**, *102* (7), 073005. https://doi.org/10.1103/PhysRevLett.102.073005.
(16) Monkhorst, H. J.; Pack, J. D. Special Points for Brillouin-Zone Integrations. *Phys. Rev. B* **1976**, *13* (12), 5188–5192. https://doi.org/10.1103/PhysRevB.13.5188.
(17) You, Z.; Hsu, C. Theory and Calculation for the Electronic Coupling in Excitation Energy Transfer. *Int. J. Quantum Chem.* **2014**, *114* (2), 102–115. https://doi.org/10.1002/qua.24528.
(18) Scholes, G. D.; Ghiggino, K. P. Electronic Interactions and Interchromophore Excitation Transfer. *J. Phys. Chem.* **1994**, *98* (17), 4580–4590. https://doi.org/10.1021/j100068a017.
(19) Krueger, B. P.; Scholes, G. D.; Fleming, G. R. Calculation of Couplings and Energy-Transfer Pathways between the Pigments of LH2 by the Ab Initio Transition Density Cube Method. *J. Phys. Chem. B* **1998**, *102* (27), 5378–5386. https://doi.org/10.1021/jp9811171.
